# Spatial mapping of the AA-PGE_2_-EP axis in multiple sclerosis lesions

**DOI:** 10.1007/s00401-025-02878-3

**Published:** 2025-04-29

**Authors:** Cathrin E. Hansen, Julia Konings, Gabor Toth, Serhii Chornyi, Manon Karsten, Bert van het Hof, Susanne M. A. van der Pol, Stephanie D. Beekhuis-Hoekstra, Nine Kok, Wing Ka Fung, Naomi S. Dijksman, Wia Baron, Maarten E. Witte, Ingela Lanekoff, Helga E. de Vries, Gijs Kooij

**Affiliations:** 1 Department of Molecular Cell Biology and Immunology, Amsterdam UMC Location Vrije Universiteit Amsterdam, De Boelelaan 1117, Amsterdam, The Netherlands; 2 Amsterdam Neuroscience, Amsterdam UMC, Amsterdam, The Netherlands; 3 MS Center Amsterdam, UMC Location VU Medical Center, Amsterdam, The Netherlands; 4 Amsterdam Institute for Immunology and Infectious Diseases, Amsterdam UMC, Amsterdam, The Netherlands; 5 Department of Chemistry, BMC, Uppsala University, 75237 Uppsala, Sweden; 6 Center of Excellence for the Chemical Mechanisms of Life, Uppsala University, Uppsala, Sweden; 7 Biomedical Sciences, Section Molecular Neurobiology, University of Groningen, UMCG, MS Center Noord Nederland, A. Deusinglaan 1, Groningen, The Netherlands

**Keywords:** Multiple sclerosis, Arachidonic acid, Prostaglandin E_2_, Mass spectrometry imaging, Neuroinflammation, Microglia

## Abstract

Bioactive lipid mediators (LMs) derived from polyunsaturated fatty acids (PUFAs) are key molecules in both the initiation and resolution of inflammatory responses. Previous findings suggest that a dysregulated LM balance, especially within the arachidonic acid (AA) pathway, may contribute to an impaired resolution response and subsequent chronic neuroinflammation in multiple sclerosis (MS). However, to date, the local biosynthesis and signaling of LMs within the brain of people with MS (PwMS) remains unexplored. In this study, we, therefore, mapped the distribution of AA and its key downstream LM prostaglandin E_2_ (PGE_2_) in white matter MS brain tissue and of non-neurological controls (NNCs) for the first time using mass spectrometry imaging. We found that AA levels are lower in MS cases compared to NNCs and reduced in MS lesions compared to peri-lesional tissue. Furthermore, the PGE_2_/AA ratio, indicating the PGE_2_ synthesis from the AA substrate, was increased in lesion areas compared to fully myelinated regions in MS. In line with that, the expression of prostaglandin synthesizing enzymes as measured by RT-qPCR was partially increased in MS tissue compared to NNCs. In addition, the expression of prostaglandin E2 receptor 4 (EP4) decreased, while prostaglandin E2 receptor 2 (EP2) showed increased expression levels in MS lesions compared to NNCs and localized specifically to microglia. We also found that PGE_2_ addition to pro-inflammatory human-induced pluripotent stem cell (iPSC)-derived microglia resulted in enhanced cytokine signaling pathways, but also the upregulation of its synthase *PTGES* and homeostatic/resolving signaling, the latter of which might mainly occur through EP2 signaling. Collectively, our results provide detailed information about the region-specific levels of AA and PGE_2_ in MS lesions and we propose enhanced PGE_2_-EP2 signaling in inflamed microglia in MS.

**Supplementary Information:**

The online version contains supplementary material available at 10.1007/s00401-025-02878-3.

## Introduction

Multiple sclerosis (MS) is a chronic inflammatory disease of the central nervous system (CNS) that manifests itself by focal demyelinating lesions within the brain and spinal cord of people with MS (PwMS) [62]. MS lesion development is temporally diverse, but typically spans weeks to months [20, 67] and is best described by a spectrum across different lesion stages [35]. In general, white matter lesions can be classified by their degree of neuroinflammation and demyelination. For example, microglia activation and clustering (termed microglia nodules) without clear signs of demyelination is suggested to predispose normal-appearing white matter (NAWM) to form pre-active lesions [9, 76, 77]. Progressing from there, active, mixed active/inactive (A/I) and chronic inactive (CIA) MS lesions all display myelin degradation but can be differentiated by their inflammatory profile (presence and activation status of microglia/macrophages). Within this profile, active MS lesions are characterized by the infiltration and accumulation of peripheral immune cells including lymphocytes and the presence of myelin phagocytosing microglia/macrophages throughout the lesion, whereas these cells are confined to the lesion border in mixed A/I lesions (hypocellular center) and almost absent in CIA [35]. To date, the local factors that drive the regional differences during MS lesion evolution remain largely elusive, and ways to further reveal the local metabolic environment that drives lesion inflammation and progression are, therefore, of high therapeutic value [27].

(Neuro)inflammation is partially orchestrated by essential polyunsaturated fatty acids (PUFAs), which are metabolized into bioactive lipid mediators (LMs) following, e.g., cytokine (e.g., TNFα or Il-1β) or reactive oxygen species stimulation [59, 61]. PUFAs include omega-3 (e.g., docosahexaenoic acid) and omega-6 FAs [e.g., arachidonic acid (AA)], which are hydrolyzed upon stimulation by cytoplasmic phospholipase A2 and converted into LMs including prostaglandins and leukotrienes [53]. Selected products of the AA pathways are elevated in the plasma of PwMS and correlate with the disease disability score [5]. Cyclooxygenases (COX1 and 2) and prostaglandin synthases convert AA to LM including prostaglandin E_2_ (PGE_2_), which is abundantly present in cerebrospinal fluid of PwMS compared to controls [19, 46], and lymphocytes increase their PGE_2_ production and secretion just before MS disease onset/symptoms occur [11]. While current disease-modifying therapies effectively modulate peripheral inflammatory episodes, the (low-grade) inflammatory state of the CNS persists. This highlights the crucial need to obtain more information on both PUFA and LM distribution in the brain and their effect on MS lesion inflammation and progression to provide insight into molecular mechanisms and more effective treatment strategies.

PGE_2_ signals via its four G-protein coupled receptors (GPCRs), termed Prostaglandin E Receptor 1-4 (EP1-4), and can evoke beneficial and/or detrimental effects [4, 31, 41, 52]. Specifically, prostaglandin E2 receptor 2 (EP2) and prostaglandin E2 receptor 4 (EP4) activation have been reported in the context of inflammatory processes such as T-cell expansion and microglia activation [15, 30, 63]. Inhibition of both receptors synergistically suppressed the development of experimental autoimmune encephalomyelitis (EAE), an animal model of MS [14]. Of note, EP2 and EP4 are both associated with G_αs_ proteins and adenylyl cyclase-cAMP signaling; however, the potential differences in their signal transduction pathways within the MS brain remain unclear.

In this study, we applied mass spectrometry imaging (MSI) to study the spatial distribution of AA and one of its key metabolites, PGE_2_, in human post-mortem brain tissue of non-neurological controls (NNCs) and PwMS. We observed lower levels of AA in MS compared to NNCs and specifically in MS lesions compared to peri-lesional tissue. Furthermore, COX2 (*PTGS2*) and prostaglandin E synthase (*PTGES*) mRNA expression were higher in MS lesion tissue blocks compared to controls, and concomitantly PGE_2_/AA is increased in demyelinated WM lesions in MS. Of note, the expression of PGE_2_ receptor EP4 decreases in MS tissue lysates, while EP2 levels increase, specifically in activated microglia. PGE_2_ signaling in pro-inflammatory human-induced pluripotent stem cell (iPSC)-derived microglia evoked extensive cytokine-receptor pathway stimulation, strengthening of PGE_2_ synthesis and initiation of homeostatic/resolving signaling, the latter mainly directed through EP2. Overall, we demonstrate a lipid distribution map in WM MS lesions that has enhanced PGE_2_-EP2 signaling, which potentially acts as a resolution mechanism to dampen neuroinflammation and thereby counteracts the initiating pro-inflammatory effects of PGE_2_ in MS.

## Materials and methods

### Human tissue samples

Post-mortem fresh-frozen (FF) and formalin-fixed paraffin-embedded (FFPE) human white matter (WM) tissue blocks were obtained from people with clinically diagnosed MS (PwMS) (total *n* = 31) and non-neurological controls (NNC) (total *n* = 19) and provided by the MS Center Amsterdam, the Normal Aging Brain Collection Amsterdam, and the Netherlands Brain Bank. Relevant clinical information and the experimental application of each donor sample are listed in Table 1 (extended version in Table S4). All donors or their next of kin had given fully informed consent for autopsy and use of material for research from the Netherlands Brain Bank under ethical approval by the Medical Ethics Committee of the Free University Medical Center in Amsterdam (2009/148), project number 1127 and project number 412 in the University of Groningen.

**Table 1:** Clinical and demographic data of MS and NNC subjects

Case ID	Age at death	Sex	PMD (h)	Cause of death	Type of MS	Tissue preservation	Lesion type	Application
NNC1	41	F	13:30	Pulmonary hemorrhage	n/a	FF	n/a	WB
NNC2	98	M	8:40	Aortic dissection	n/a	FF	n/a	WB
NNC3	82	M	13:35	Heart failure	n/a	FF	n/a	WB
NNC4	53	M	14:25	Cardiac complications	n/a	FF	n/a	WB
NNC5	78	M	5:55	Cardiac failure	n/a	FF	n/a	WB
NNC6	77	F	9:15	Sudden death	n/a	FF	n/a	WB
NNC7	88	F	5:40	Cardiac failure	n/a	FF	n/a	WB
NNC8	77	F	19:45	Malignant lymphoma	n/a	FF	n/a	WB
NNC9	91	F	5:45	Sudden death	n/a	FF	n/a	WB
NNC019	68	M	8:40	Euthanasia	n/a	FF, FFPE	n/a	MSI, IHC (COX2, EP2), mRNA
NNC095	68	M	7:35	Esophageal carcinoma, narrowing aa. coronaries	n/a	FFPE	n/a	IHC (EP2)
NNC880	77	M	11:25	Pneumonia	n/a	FFPE	n/a	IHC (EP2)
NNC945	59	F	8:10	Euthanasia, terminal COPD	n/a	FF	n/a	MSI, IHC(COX2), mRNA
NNC989	71	F	7:50	Lung carcinoma	n/a	FF	n/a	MSI, IHC (COX2), mRNA
NNC999	74	M	10:20	Euthanasia	n/a	FF	n/a	IHC (COX2), mRNA
NNC227	93	M	6:00	Cachexia, cerebrovascular accident/kidney failure	n/a	FF	n/a	mRNA
NNC254	75	F	5:40	Cachexia	n/a	FF	n/a	mRNA
NNC275	82	F	5:10	Pneumonia by hemothorax	n/a	FF	n/a	mRNA
NNC215	79	M	9:00	Pneumonia and metastasized kidney carcinoma	n/a	FF	n/a	mRNA
MS1	70	F	6:30	Probably urosepsis	SPMS	FF	unk	WB
MS2	53	F	10:45	Euthanasia	SPMS	FF	unk	WB
MS3	48	F	4:50	Euthanasia	SPMS	FF	unk	WB
MS4	48	F	8:10	Euthanasia	SPMS	FF	unk	WB
MS5	75	F	8:00	Pneumonia	SPMS	FF	unk	WB
MS6	43	M	8:30	Pneumonia	SPMS	FF	unk	WB
MS7	53	M	5:53	Pneumonia	PPMS	FF	unk	WB
MS8	52	F	8:25	Pneumonia	PMS	FF	unk	WB
MS9	48	F	5:50	Congestive cardiac failure	SPMS	FF	unk	WB
MS10	66	F	6:20	Cancer	SPMS	FF	unk	WB
MS11	71	F	10:15	Post-surgery respiration problems	PMS	FF	unk	WB
MS12	64	M	7:30	End stage prog. MS	PPMS	FF	unk	WB
MS13	65	M	10:35	Urosepsis	SPMS	FF	unk	WB
MS14	77	M	4:15	Cerebral vascular accident	PPMS	FF	unk	WB
MS68	48	F	9:20	Pneumonia	unk	FF	AI/I	MSI, IHC (COX2), mRNA
MS46	51	F	9:10	Euthanasia	SPMS	FF, FFPE	A	MSI, IHC (COX2, EP2), mRNA
MS71	67	M	7:55	Euthanasia	unk	FFPE	A/I	IHC (EP2)
MS85	67	F	11:25	Pneumonia	unk	FFPE	A/I	IHC (EP2)
MS16	77	F	9:45	Aspiration pneumonia	SPMS	FF, FFPE	A/I	MSI, IHC (COX2, EP2), mRNA
MS31	53	F	5:50	Euthanasia	PMS	FFPE	A/I	IHC (EP2)
MS60	60	F	5:05	Euthanasia	SPMS	FF	A/I	MSI, IHC (COX2), mRNA
MS42	82	F	7:30	Cardiac arrest, sudden death	PPMS	FF	A/I	MSI, IHC (COX2), mRNA
MS32	54	M	unk	Progressive dyspnea	unk	FF	A	MSI, IHC (COX2), mRNA
MS116	66	F	9:30	Euthanasia	SPMS	FF	CIA	MSI, IHC (COX2), mRNA
MS120	73	M	8:45	Urosepsis	SPMS	FF	A/I	mRNA
MS100	71	F	7:05	Cachexia with PMS and metastatic breast cancer	PMS	FF	A/I	mRNA
MS298	53	F	10:45	Euthanasia	PMS?	FF	unk	mRNA
MS51	47	M	7:15	Urosepsis with organ failure	SPMS	FF	unk	mRNA
MS139	56	M	8:00	Pneumonia	SPMS	FF	unk	mRNA

PMD post−mortem delay, unk unknown, n/a not applicable, mRNA messenger RNA, PMS progressive MS, PPMS primary progressive MS, SPMS secondary progressive MS, FF fresh frozen, FFPE formalin−fixed paraffin−embedded, CIA chronic inactive, A/I mixed active/inactive, A active, MSI Mass Spectrometry Imaging

### Mass spectrometry imaging

FF human tissues were cryo-sectioned (CryoStar NX70, Thermo Fisher Scientific, Waltham, USA) into 10 µm, thaw-mounted onto super-frost microscopy glass slides, and stored at − 80 °C until further use. Before mass spectrometry imaging (MSI), excess salt was removed from the gently thawed tissue sections by immersing the slides for 1 min six times in Milli-Q water (18.2 MΩ) and drying the slides at room temperature under N2 flow. Silver-doped pneumatically assisted nanospray desorption electrospray ionization (PA nano-DESI) MSI was performed as previously described [12]. Briefly, the PA nano-DESI probe comprised two fused silica capillaries (50 µm inner diameter, 150 µm outer diameter) placed at ca. 90° angle to each other. A liquid bridge was formed at the intersection of the primary and the secondary capillary by delivering the extraction solvent through the primary capillary with a syringe pump (KD Scientific, Holliston, USA) at a flow rate of 0.5 µL/min. The extraction solvent was composed of acetonitrile:methanol v/v 9:1 (0.1% formic acid) spiked with 10 ppm ^107^Ag^+^ and internal standards (1 µM Prostaglandin E2 (PGE_2_)-d9, 1 µM arachidonic acid (AA)-d8) for normalization and quantitation [13]. Pneumatic assistance was provided by co-axial nitrogen flow to the tip with a gas pressure of 3.5 bar.

The tissue-containing slides were moved under the probe with an XYZ stage (Zaber Technologies, Canada) controlled by an in-house developed LabView program [37]. Lines were acquired by scanning at 50 µm/s in the X direction and stepping 150 µm in the Y direction, thus generating pixels of ~ 25 × 150 µm. The experiments were performed on an IQ-X mass spectrometer (Thermo Fischer Scientific) operated at a capillary voltage of 3.5 kV, and a heated capillary temperature of 275 °C. Data were acquired by alternating full scan (m/z 200–1100) and targeted selected ion monitoring (tSIM) (m/z 425–475) scan events with a resolution of 120 000 (at m/z 200), and the orbitrap automatic gain control target was set to 50% and 40%, respectively. Ion images were created with the in-house developed ion-to-image (i2i) application [42] using 5 parts per million (ppm) mass tolerance and an automatic contrast adjustment was applied to the 99th percentile of intensities. Quantitative ion images were generated in i2i by normalizing endogenous intensities pixel-by-pixel to the respective internal standard and ratio images by normalizing two endogenous signals.

#### Region selection and quantification

Consecutive brain sections of the tissues visualized by PA nano-DESI MSI were stained for myelin proteolipid protein (PLP) and Human Leukocyte Antigen–DR isotype (HLA-DR) to classify the amount of (de)myelination and inflammation in NNC and MS tissue. To this end, full tissue optical scans at 20 × magnification were obtained with the Olympus VS200 slide scanner (Evident, Tokyo, Japan). Brain tissue areas were created and stratified based on PLP and HLA-DR stainings using QuPath software (version 0.4.4) along with visually prominent intensity differences in ion images generated with PA nano-DESI MSI. The details of lesion classification are outlined in the Results section [21, 35]. For analysis of the *in-depth* classification, ROIs with the same PLP or HLA-DR classification were averaged per donor. ROIs were transferred into the i2i application [42] and quantities were extracted for each lipid of interest using one-point calibration. Absolute values (µM/pixel) and relative values (PGE_2_ /AA ratio) were used. Statistical analysis was performed with GraphPad Prism version 10.2.

### Human iPSC-derived microglia

Human-induced pluripotent stem cells (hiPSC) were generated from NNC, with the approval of the LUMC scientific ethical committee, and obtained informed consent (NL45478.058.13/P13.080) [6]. The generation of iPSC-derived microglia (hiPSC microglia) was performed according to the protocol of Kenkhuis et al. and previously reported [21, 32]. In short, iPSCs (cell line 114-1, female, 49 years) were used to develop mesodermal embryoid bodies, which were plated and supplemented with medium containing macrophage colony-stimulating factor (rhM-CSF, 100 µg/ml; 300-25, Peprotech) and Interleukin 3 (25 µg/ml; #200-03-B, Peprotech). Half of the media was refreshed weekly for 3–4 weeks and once mEBs started producing myeloid precursor cells, they were harvested weekly. To obtain mature microglia, the harvested myeloid precursor cells were plated in plates coated with 0.01 g/ml poly-D-lysine (Sigma, Saint Louis, MI, USA) and 0.01% gelatin type B (Sigma, Saint Louis, MI, USA) and cultured for 2 weeks in Advanced DMEM/F12 (Invitrogen, Waltham, MA, USA) supplemented with 100 U/mL penicillin–streptomycin (Invitrogen, Waltham, MA, USA), 2 mM Glutamax (ThermoFisher, Walthman, MA, USA), 50 µM 2-β-mercaptoethanol (ThermoFisher, Walthman, MA, USA), N2 supplement (ThermoFisher, Walthman, MA, USA), 2 µg/ml recombinant human GM-CSF (Peprotech, Rocky Hill, NJ, USA), and 20 µg/ml recombinant human IL-34 (Peprotech, Rocky Hill, NJ, USA). The phenotypic identity of the differentiated cells was confirmed by the immunocytochemistry analysis of microglia markers (Iba1, P2RY12, TMEM119). The acquired matured iPSC microglia were either used untreated (resting) or were subjected to treatment with lipopolysaccharide (LPS) (100 ng/ml; L2630, Sigma-Aldrich) and IFNγ (20 ng/ml; #300–02, Peprotech) (pro-inflammatory) for 24 h. For every batch, cell phenotypes were assessed by qPCR analysis. Subsequently, cells were washed twice with PBS and treated with PGE_2_ (1 μM; P0409, Sigma-Aldrich) or vehicle (0.1% ethanol) for 24 h in serum-free medium. In addition, selective EP2 (10 μM; TG6-10-1, Calbiochem) and EP4 receptor inhibitors (1 μM; ONO AE3 208, Tocris) or vehicle (DMSO) were applied, together with the PGE_2_ treatment. For RNA sequencing applications, hiPSC microglia (1 biological, 5 technical replicates per condition) were washed with PBS and lysed in 275 µl Buffer RLT Plus (#1053393; Qiagen), after which they were scraped, collected, and stored at − 70 °C until further use.

### Immunostainings

For immunostainings, FF or FFPE human WM tissue blocks were sliced in 5 μm sections, dried or deparaffinized, and washed with Milli-Q water (Millipore). FFPE sections were subjected to heat-mediated antigen retrieval for 10 min (10 mM sodium citrate buffer, pH 6) and then brought to room temperature on ice for 30 min. FF sections were fixed with 4% PFA for 10 min at room temperature. All sections were washed with PBS and blocked with PBS containing 10% bovine serum albumin (BSA, Fraction V, Roche Diagnostics) or normal species serum (NSS) and 0.05% Tween20 (Sigma-Aldrich) for 30 min. Primary antibodies for EP2 (1:200; Abcam, ab124419), Iba1 (1:500; Abcam, ab5076), TMEM119 (1:50; R&D systems, MAB10313), COX2 (1:100; Cayman Chemical. 20,198), CD45 (1:200; Abcam ab10559,), Collagen IV (1:300, Invitrogen 51-9871-82), HLA-DR (1:500; Hybridoma), PLP (1:300; Serotec, MCA839G), and UEA-I (1:1000, Vector Labs B-1065) were diluted in PBS containing 1% BSA/NSS and 0.05% Tween20, and incubated in the dark overnight at 4 °C. Sections were washed with PBS and incubated with the Alexa fluorophore-conjugated secondary antibody diluted in PBS containing 0.05% Tween20, for 1 h at room temperature in the dark. For nuclear staining, Hoechst (33,258, Thermo Fisher Scientific) was diluted in PBS to a final concentration of 10 μg/mL and applied for 1 or 7 min in the dark. Partially, sections were submerged in Sudan Black B (0.03% in 70% EtOH) for 10 min to reduce autofluorescence. Lastly, sections were washed with PBS, mounted with Mowiol and a coverslip (Menzel-Glaser, thickness #1), and stored in the dark at 4 °C until image acquisition.

### Microscopy and image acquisition

**Fig. 1:**
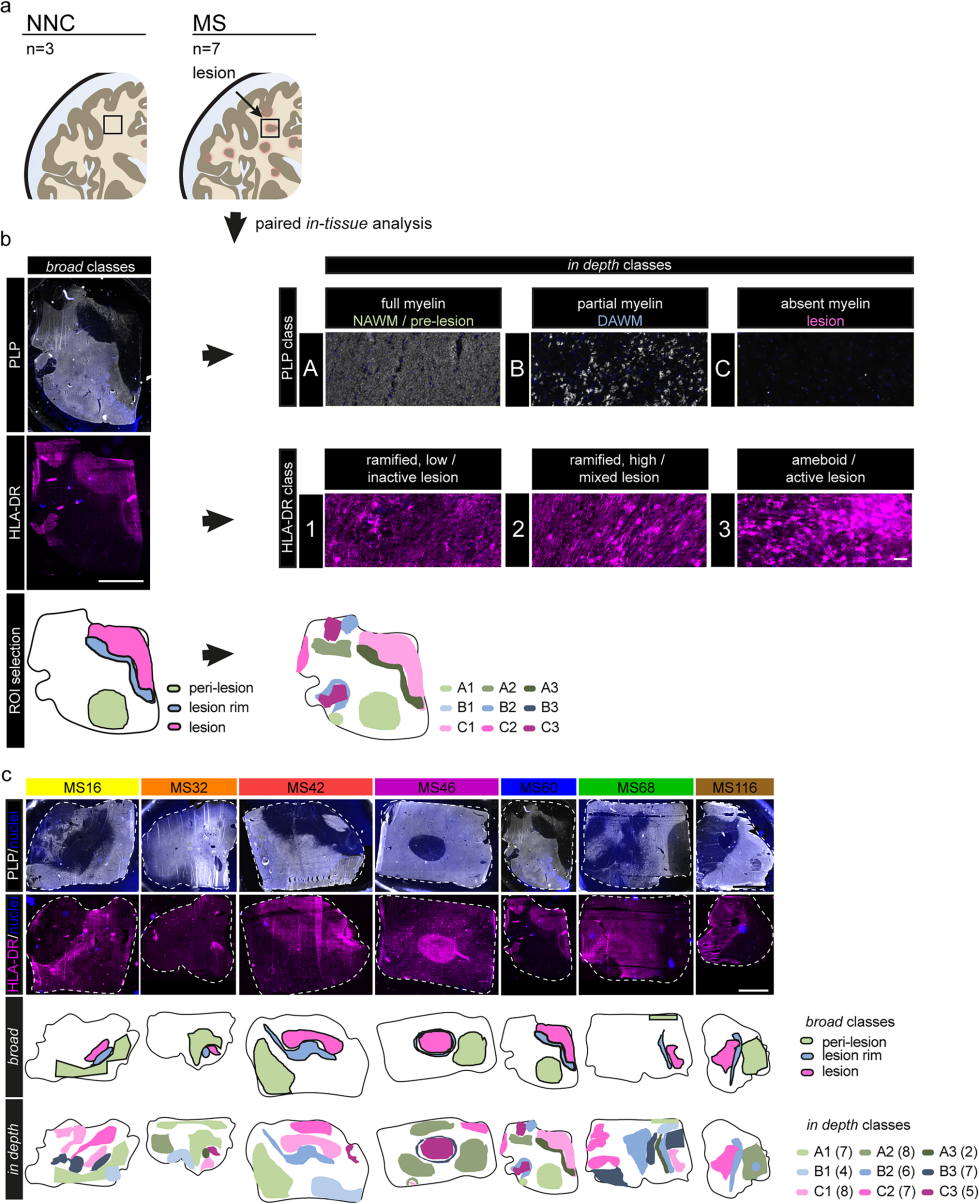
Broad and in-depth tissue classification of NNC and MS human brain tissue. **a** Study sampling of three NNCs and seven MS human post-mortem brain tissues. **b** ROIs were defined by differences in neuropathology, assessed by PLP and HLA-DR reactivity. Within the MS tissues, *broad* tissue classes encompass a representative ROI for a lesion, adjacent lesion rim and distant peri-lesional tissue; scale bar: 5 mm. For *in-depth* quantification, ROIs were stratified by PLP reactivity (**A** full, **B** partial and **C** absent/demyelinated), and HLA-DR density and morphology (1: ramified, low density and inactive lesion; 2: ramified, high density, mixed active/inactive lesion; 3: rounded, ameboid, high density and active lesion); scale bar: 50 µm **c** Immunohistochemical depiction of PLP and HLA-DR in MS tissues (color-coded) and annotated ROIs for *broad* and *in-depth* tissue classification; scale bar: 5 mm.

EP2-Iba1-TMEM119 immunoreactivity was assessed with a 10 × overview scan, after which ROIs were imaged with a 40× air objective using the Akoya Vectra Polaris Automated Quantitative Pathology Imaging System. Images were spectrally unmixed using inForm (version 3.0, Akoya Biosciences, Menlo Park, CA) and batch-analyzed using NIS elements (version 5.30.03, Nikon Europe B.V., Amsterdam, The Netherlands). Cells were defined by the overlap of nuclear and marker staining, and cell counts plus mean fluorescent intensity were extracted. Images of PLP, HLA-DR, COX2, CD45, UEA-I, and CollagenIV immunoreactivity were taken at the Olympus VS200 slide scanner using a 20 × overview scan and 60 × images (Olympus X line, 1.42 NA, oil) within the respective ROIs used previously for lipid quantification (Fig. 1c). Images were deconvolved using Huygens Professional 21.10 software (scientific volume imaging B.V.) and (if possible) batch-analyzed using NIS elements. The mean fluorescent intensity of COX2 was measured in an enlarged nuclear mask in CD45^+^ cells.

### Western blot

FF human brain tissue was cryo-sectioned and lesions were roughly excised by needle excision (yielding 50 µg tissue) [71]. Tissue was homogenized with a douncer on ice in 10 mM Tris (pH 7.4), 2 mM EDTA, 0.25 M sucrose, and a mix of protease inhibitors and stored at − 80 ºC until use. hiPSC microglia were washed, lysed on ice in RIPA buffer for 15 min, scraped from the plate, and lysates were stored at − 20 ºC until further use. Western blots were performed similarly to previous work [29]. In short, samples were further diluted in Laemmli Buffer (#161-0747; Bio-rad 4x), after which they were heated to 95 °C for 5 or 10 min, centrifuged, and applied on a 10% or 12% SDS-PAGE. After transfer to a nitrocellulose (cell lysate samples) or PVDF membrane (tissue homogenate samples), the blots were incubated for 1 h at room temperature in blocking solution intercept (TBS) blocking buffer (927-60001, LI-COR) diluted 1:1 in TBS containing 0.1% Tween20. Blots were incubated with EP2 (1:250; Abcam; ab124419), and EP4 (1:100, Santa Cruz Biotechnology; sc-55596) antibodies diluted in blocking solution overnight at 4 °C. After washing with TBS containing 0.1% Tween20, IRDye secondary antibodies were incubated for 1 h at room temperature. Blots were washed with TBS containing 0.1% and visualized by Azure Sapphire Biomolecular Imager for cell lysates and Odyssey Clx Imaging System for tissue homogenates. β-Actin (1:5000; Sigma-Aldrich; A5441) and α-tubulin (1:5000; Cedarlane; CLT9002) were used as loading control for sample normalization. Densitometric analysis was performed with ImageJ (version 1.49v) for cell lysates and Image Studio Lite software (version 5.2) for tissue homogenates. Raw blots are added in Additional File 1.

### RNA isolation and real-time quantitative polymerase chain reaction (qRT-PCR)

After treatment, hiPSC microglia were washed with PBS, and RNA was extracted using TRIzol (#15596-018, Thermo Fisher Scientific), whereas RNA from human brain tissue blocks was extracted using the RNeasy Lipid Tissue Mini Kit (#174804, Qiagen). Total RNA quantity was evaluated using a Nanophotometer (Implen, Westlake Village, USA) and cDNA was synthesized using the High-Capacity cDNA Reverse Transcription Kit (#4368813, Thermo Fisher Scientific). Transcripts of interest were visualized and measured using SYBR Green (#4309155, Thermo Fisher Scientific) and the QuantStudio^™^ 3 Real-Time PCR System (#A28567, Thermo Fisher Scientific). Expression was measured in experimental and technical replicates and the 2 − ^ΔΔ^CT relative quantification method was applied to normalize the expression to the housekeeping genes *Polrf2* (hiPSC microglia) and *β-actin* (human brain tissue block lysates). Primer sequences are summarized in Table 2

**Table 2:** Primer details

Target	Forward primer (5'–3')	Reverse primer (5'–3')
ACTB	GGG AAA TCG TGC GTG ACA TTA AG	TGT GTT GGC GTA AGG TCT TTG
POLRF2	GAA CTC AAG GCC CGA AAG	TGA TGA TGA GCT CGT CCA C
PTGER2	TGC CTT TCA CGA TTT TTG CA	ACG CAT TAG TCT CAG AAC AGG A
PTGER4	TACTCATTGCCACCTCCCTGGT	GACTTCTCGCTCCAAACTTGGC
PTGS1	TCT TGC TGT TCC TGC TCC TG	GTC ACA CTG GTA GCG GTC AA
PTGS2	ACA GGC TTC CAT TGA CCA G	TCA CCA TAG AGT GCT TCC AAC
PTGES	CAC CGG AAC GAC ATG GAG A	TCC AGG CGA CAA AAG GGT TA
PTGES2	AGC AAG CGA CTC AAG AGC AG	GCC ATA CAG CGC CAA ATC AG
PTGES3	TGC CCC GTT CAC AAT ACT CC	CAG GCT GCT TTT CCA AAG ACA T

### RNA isolation and Quant Seq 3′mRNA-Seq

Lysates were thawed on ice, after which RNA was extracted using the RNeasy Plus Micro kit (#74034; Qiagen). Obtained RNA concentrations were measured using the Qubit® 3.0 Fluorometer (ThermoFisher), and the Quant Seq 3′ mRNA-Seq V2 Library Prep Kit FWD with Unique Dual Indices (#UDI12A_0001-0096; Lexogen) was used according to manufacturer’s instructions to prepare the sequencing libraries. Quality control was assessed using the 4200 TapeStation (Agilent Technologies). Sequencing libraries were pooled equimolarly, and sequenced was performed on a NextSeq 2000 (Illumina). Sequencing data were pre-processed by Lexogen Bioinformatics Services (Vienna, Austria) using their in-house pipeline, which includes quality control and adaptor trimming, alignment to the most recent human reference genome (homo_sapiens_GRCh38_ensembl_release_10 7_ERCC_SIR), and PCR deduplication trough unique molecule identifier (UMI) processing.

#### Differential expression analysis

Downstream bioinformatics was applied in R (v4.3.2) using RStudio (v2023.12.1) [73, 74]. Data processing and normalization and subsequent differential expression (DE) analysis were executed using the edgeR (v4.0.16) and limma (v3.58.1) packages [7, 65]. Principal component analysis (PCA) plots visualizing sample clustering across conditions were generated using ggplot2 (v3.5.1) [79]. Differentially expressed genes (DEGs) were based on an adjusted p-value of less than 0.05 and a Log|FC| greater than 1, which were depicted in Venn diagrams and volcano plots produced using the ggvenn (v0.1.10) and EnhancedVolcano (v1.20.0) packages [39, 82]. Over-representation analysis (ORA) was used to identify Kyoto Encyclopedia of Genes and Genomes (KEGG) terms enriched based on the DEG lists, using the clusterProfiler (v.4.4.4) package [80].

### Statistical analysis

Data are shown as boxplots displaying minimum to maximum values, with the middle line representing the median. All statistical tests (except RNA seq. data) were performed in GraphPad Prism v10.02 (GraphPad Software, La Jolla, USA) and based on at least three independent experiments. In the case of a sample size > 10, outliers were identified and removed using Grubb’s method (*α* = 0.05), which was only the case in MS lesion tissue block lysate RT-qPCR samples. Data normality was assessed using the Shapiro–Wilk test. In multiple (> 2) group comparisons, we used a two-tailed one-way analysis of variance (ANOVA) with Tukey’s/Dunnett’s (paired) multiple comparisons test. Friedman test (paired) or Kruskal–Wallis test was used for non-normally distributed data followed by Dunn’s post hoc analysis. For comparing two experimental groups, we used a two-tailed Student’s t-test or a Mann–Whitney test in the case of non-parametric data. To correlate lipid data with pathological hallmarks, we used Spearman’s rank correlation coefficient (non-parametric data). Analysis details are stated in figure legends, accompanied by corresponding p-values, of which *p* < 0.05 (red) was considered statistically significant.

## Results

### Classifications of NNC and MS white matter brain tissue

To provide a spatial distribution map of AA and PGE_2_ in MS lesions, FF human brain tissues from NNCs (*N* = 3) and MS lesions (*N* = 7) (Fig. 1a) were used to stratify MS brain tissue, using PLP and HLA-DR immunoreactivity to reveal the level of myelination and neuroinflammation, respectively. Based on these histological markers, we created two separate classifications for the MS tissue to allow for a general (“*broad*”) and a more differentiated (“*in-depth*”) analysis of the LM profile in MS brain tissue. A paired analysis within these classifications was performed in the MS tissues to provide tissue comparisons. For the *broad* classification, three regions of interest (ROIs) per donor, corresponding to the lesion, lesion rim, and peri-lesion (distant from the lesion), were chosen based on one selected lesion within the tissue (Fig. 1b,c). For the *in-depth* tissue classification, PLP reactivity was used to classify multiple areas into “A—fully myelinated (full)” (representing control brain tissue, normal-appearing white matter (NAWM), pre-lesion), “B—diffusely myelinated/partially demyelinated” (dirty-appearing WM (DAWM)), and “C—absent myelin/completely demyelinated” (MS lesions) (Fig. 1b, c). In addition, HLA-DR reactivity was stratified by cell morphology and quantity (assessed visually) into “ramified, low density (1)”, “ramified, high density (2)” and “rounded, ameboid (3)”. For lesions specifically, the terms active (high density and ameboid), mixed active/inactive (medium density, rounded), and inactive (low density, ramified to rounded) were used based on previously used lesion classifications. The *in-depth* classification allows us to cluster multiple regions within the tissues and highlight potential differences based on both the level of myelination and inflammation. Selected ROIs per donor for *broad* and *in-depth* classification are depicted in Fig. 1c.

### Arachidonic acid levels decrease in MS lesions

**Fig. 2:**
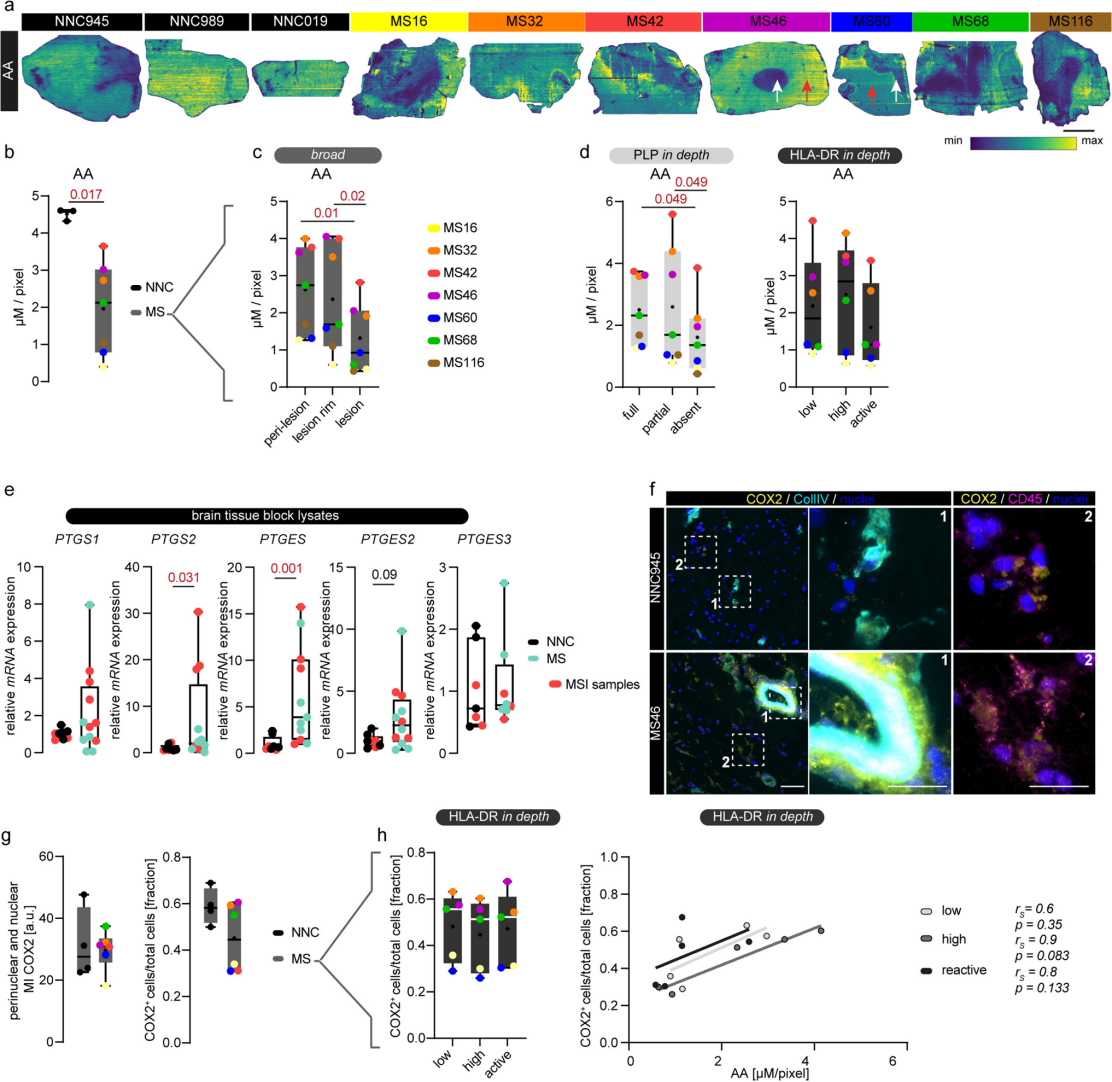
Decreased AA levels in WM MS lesions. **a** Ion images of AA [M + 107Ag] + (m/z 411.1448 ± 5 ppm) normalized to internal standard AA-d8 in human NNC (*n* = 3) and MS brain tissues (*n* = 7). The spatial distribution of AA is visualized by a min–max intensity scale within each ion image; hence, the images are not related to each other. White arrows point out exemplary demyelinated/lesion areas; red arrows indicate myelinated/peri-lesional tissue. Pixel size is 25 µm × 150 µm; scale bar: 5 mm. **b** AA signal intensities from the entire tissue section were extracted and normalized to the internal standard AA-d8 to yield the average detected concentration per pixel (µM/pixel). Each dot represents the tissue of one donor. **c** Paired analysis of AA within MS tissues based on *broad* classification (one representative ROI per tissue area). Each colored dot represents the tissue of one donor. **d** Paired analysis of AA levels based on *in-depth* classification based on PLP or HLA-DR (averages of multiple ROIs per class). Each colored dot represents the tissue of one donor. **e** *mRNA* expression of various biosynthesizing enzymes in human brain tissue block lysates of NNC (*N* = 8) and MS tissues (*N* = 12). Tissues used both in MSI and qPCR are marked in red. **f** Representative images of COX2, CD45 (immune cell marker), and Collagen IV (Coll IV, vascular marker) immunoreactivity in MS WM tissue; scale bar: 50 µm, zoom in: 25 µm. **g** Quantification of COX2 mean fluorescent intensity (MI) measured within the nuclei and percentage of COX2^+^ cells in NNC (*N* = 4) and MS lesions (*N* = 6). **h** Paired tissue analysis of COX2^+^ cells in MS tissues (HLA-DR *in-depth*) and Spearman correlation (*r*_*s*_) of detected AA concentration with COX2^+^ cells (HLA-DR *in-depth*). Data is shown as box plots with median ± quartiles; whiskers extend to minimum and maximum. Data have been statistically tested for three groups by Friedman test (paired) for non-normally distributed data and Dunn’s post hoc analysis. For two groups, an unpaired student t-test with Welch’s correction was used when the variance of the groups was significantly different or the Mann–Whitney test for non-parametric datasets. Exact p-values are reported and statistical significance is set at *p* < 0.05 (red)

AA is the key substrate for a plethora of LMs including prostaglandins, leukotrienes, and lipoxins through enzymatic and non-enzymatic reactions [16]. We performed silver-doped PA nano-DESI MSI for AA, where ion images show regional heterogeneity of AA levels in NNC tissues, but profoundly sharp outlined differences within MS lesion tissues (Fig. 2a). In particular, MS lesion areas characterized by demyelination (exemplified by white arrows) reveal lower AA intensities compared to myelinated regions within the same tissue section (exemplified by red arrows) (Fig. 2a). The mean detected AA concentrations per donor are consistently lower in MS compared to NNCs (*p* = 0.017) (Fig. 2b). Moreover, paired analysis within the MS tissue sections revealed significantly lower AA concentrations in the lesion area compared to the peri-lesion area (*p* = 0.01) (*broad* classes; Fig. 2c), and between lesion rim and lesions (*p* = 0.02), whereas no differences in AA were observed between the lesion rim and peri-lesional areas (Fig. 2c). *In-depth* classification showed that AA levels were significantly lower in demyelinated areas (absent) compared to both full and partial myelination (*p* = 0.049; *p* = 0.049) (*in-depth* classes; Fig. 2d). The different inflammatory states (low, high, active) did not show changes in AA (*in-depth* classes; Fig. 2d). Essential fatty acids, including AA, are transported from the blood circulation across brain endothelial cells into the CNS [60]. We observed that the vascularized area, assessed by endothelial marker Ulex Europaeus Agglutinin I (UEA-I), was similar between full, partially demyelinated and demyelinated areas (Fig. S1a, b). Interestingly, detected AA concentrations correlate positively with the vascularized area in fully myelinated tissue (*p* = 0.017, *r*_*s*_ = 1) and trend to correlate with demyelinated areas (*p* = 0.103, *r*_*s*_ = 0.771), but not in partially demyelinated regions (*p* = 0.241, *r*_*s*_ = 0.6) (Fig. S1c).

Decreasing AA concentrations in MS tissue and MS lesions could point towards increased metabolism of AA-derived LMs [78]. Hence, we next quantified the expression of biosynthesizing enzymes like cyclooxygenases and prostaglandin synthases involved in the AA to prostaglandin conversion in human tissue block lysates by RT-qPCR (Fig. 2e) [64]. Transcripts for *PTGS2* (encoding for COX2) and *PTGES* (encoding for mPGES-1) were significantly higher in MS compared to NNCs (*p* = 0.031; *p* = 0.001, respectively), and *PTGES2* (encoding for mPGES-2) showed a similar trend (*p* = 0.09), whereas *PTGS1* (encoding for COX1) and *PTGES3* (encoding for cytosolic PGES) were not differently expressed between MS and NNCs (Fig. 2e). Next, we visualized the rate-limiting enzyme for PGE_2_ biosynthesis, COX2, by immunohistochemistry (IHC) in consecutive brain slices to the MSI-imaged tissue sections [45]. We found COX2 immunoreactivity predominantly in larger vessels (panel 1, Fig. 2f) but predominantly around numerous nuclei expressing CD45 (panel 2), which we confirmed using confocal imaging (Fig. S1e), both in NNC and MS cases (Fig. 2f). Sporadically, we observed COX2 expression in CD45^+^ cells within the perivascular space in MS cases (Fig. S1d) [66, 81]. However, quantification of COX2 mean intensity (MI) perinuclear and percentage of COX2^+^ cells (of total cells) did not differ between NNC and MS (Fig. 2g). Moreover, within MS tissues, the number of COX2^+^ cells did not alter between the different inflammatory states. However, at both high and active states of HLA-DR^+^ cells, COX2^+^ cells tend to positively correlate with the AA concentration in the same region (*r*_*s*_ = 0.9, *p* = 0.083; *r*_*s*_ = 0.8, p = 0.133 for the *in-depth* classes respectively) (Fig. 2h). Collectively, these findings point towards an overall decrease of AA concentration in MS WM brain tissues compared to NNCs, including an MS lesion-specific decrease compared to surrounding tissue. Together with the (partial) increase in biosynthesizing enzymes, this might suggest a local activation of AA metabolism within MS brain tissue.

### Prostaglandin E_2_/AA ratio increases in MS lesions

**Fig.3:**
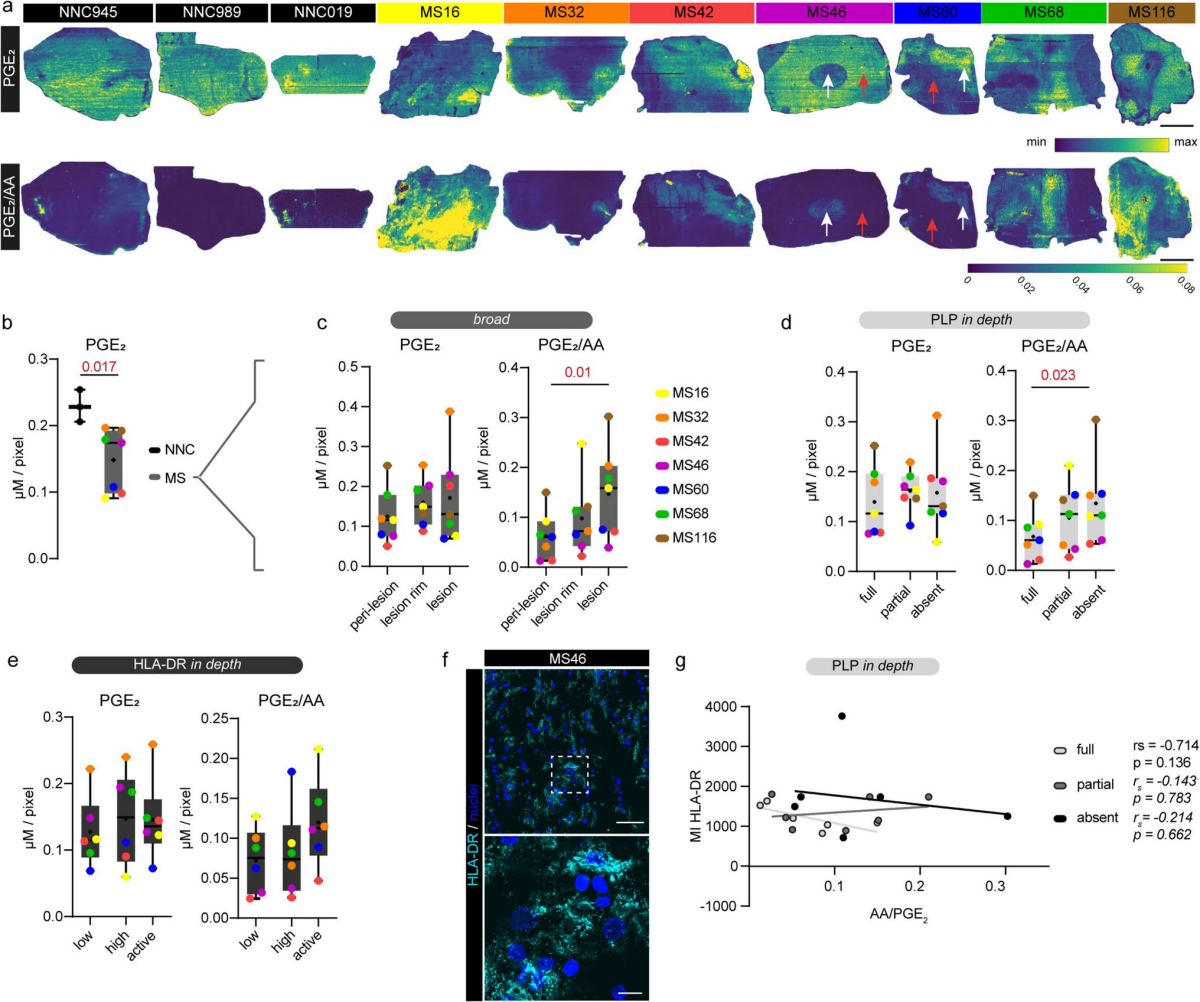
PGE_2_/AA levels are increased in demyelinated WM MS tissue. **a** Ion images of PGE_2_ (first row) and PGE_2_/AA concentration ratio images (second row). PGE_2_ ion images were constructed by visualizing *m/z* 459.1301 ± 5 ppm ([M + ^107^Ag]^+^) normalized pixel-by-pixel to internal standard PGE_2_-d9 in human NNC (*n* = 3) and MS lesions (*n* = 7). The spatial distribution of PGE_2_ is visualized by a min–max intensity scale within each single ion image; hence, the images are not related to each other. In contrast, all the PGE_2_/AA concentration ratio images are fitted to the same scale and can be compared. White arrows point out exemplary lesion areas; red arrows indicate peri-lesional tissue with full myelination; scale bar: 5 mm. **b** PGE_2_ signal intensities were extracted as average pixel intensity per tissue and normalized to the internal standard of PGE_2_ to yield average detected concentration per pixel (µM/pixel). Each dot presents one donor. **c** Paired analysis of PGE_2_ concentration and PGE_2_/AA concentration ratio based on *broad* classification (one representative ROI) within MS tissues. Each colored dot represents the tissue of one donor. **d**, **e** Paired analysis of PGE_2_ and PGE_2_/AA ratio based on PLP or HLA-DR classification (*in-depth* classes). Each colored dot represents the tissue of one donor. **f** Representative images of HLA-DR immunoreactivity in MS WM tissue; scale bar: 50 µm, zoom in: 10 µm. **g** Spearman correlation (*r*_*s*_) of PGE_2_/AA ratio values with HLA-DR MI in present, partial, and absent PLP tissue categories. Data is shown as box plots with median ± quartiles; whiskers extend to minimum and maximum. Data have been statistically tested for three groups by Friedman test (paired) for non-normally distributed data and Dunn’s post-hoc analysis. Exact p-values are reported and statistical significance is set at *p* < 0.05 (red)

The observed increased expression of some prostaglandin synthesizing enzymes, particularly *PTGES,* in MS tissues suggests an increased prostaglandin biosynthesis. To verify this hypothesis, we next focused on the distribution of the bioactive LM prostaglandin E_2_ (PGE_2_) using PA nano-DESI MSI and report both the detected PGE_2_ concentrations as well as the PGE_2_/AA ratio (Fig. 3a). Similar to the spatial distribution of AA, PGE_2_ delineated lesion areas within the MS tissues, while being quite homogenously distributed in the NNC tissues (Fig. 3a). Interestingly, some MS lesions (white arrow) showed reduced PGE_2_ levels compared to peri-lesional tissue (red arrow), while others showed a distinct increase of PGE_2_ in the lesion (Fig. 3a, MS46 and MS60 tissue, respectively). The PGE_2_/AA concentration ratio uniquely indicates the PGE_2_ concentrations relative to the availability of AA substrate [8, 40]. PGE_2_/AA ratio images reveal that increased relative PGE_2_ was generally restricted to MS lesions and partially to the adjacent tissue, except for MS68, where lesions showed lower PGE_2_/AA ratio compared to the remaining tissue (Fig. 3a, comparable scale between images). In general, whole-tissue PGE_2_ levels were lower in MS compared to NNCs (Fig. 3b).

Paired analysis of PGE_2_ concentrations within the MS tissue varied widely and did not follow a pattern within the immunohistochemistry-derived classifications of *broad* or *in-depth* (Fig. 3c–e, first graph, respectively). However, the PGE_2_/AA ratio was increased in the lesion tissue compared to peri-lesional tissue (*p* = 0.01) in the *broad* tissue classification (Fig. 3c) and also significantly higher (*p* = 0.023) in demyelinated regions compared to fully myelinated regions (*in-depth* classes; Fig. 3d). No difference was observed between partially demyelinated and fully demyelinated areas (*in-depth* classes; Fig. 3d). Categorized by HLA-DR, PGE_2_/AA concentration ratio did not significantly differ between the groups (*in-depth* classes; Fig. 3e).

We observed that the PGE_2_/AA ratio pattern partly co-localized with the HLA-DR reactivity in the same tissue (Fig. S2). Hence, we quantified the MI of HLA-DR (Fig. 3f) and correlated it with the PGE_2_/AA ratio within the PLP classes (Fig. 3g). None of the correlations reached significance (partial *r*_*s*_ = − 0.143, *p* = 0.783; absent: *r*_*s*_ = − 0.214, *p* = 0.662) and PGE_2_/AA ratios in full myelinated regions showed a negative trend with immune cell activation (*r*_*s*_ = − 0.714, *p* = 0.136). Together, these findings suggest that the PGE_2_/AA ratio increases in MS lesions but no clear association between PGE_2_/AA levels and immune cell activation (MI HLA-DR) could be made.

### Enhanced microglial EP2 expression in MS lesions

**Fig. 4:**
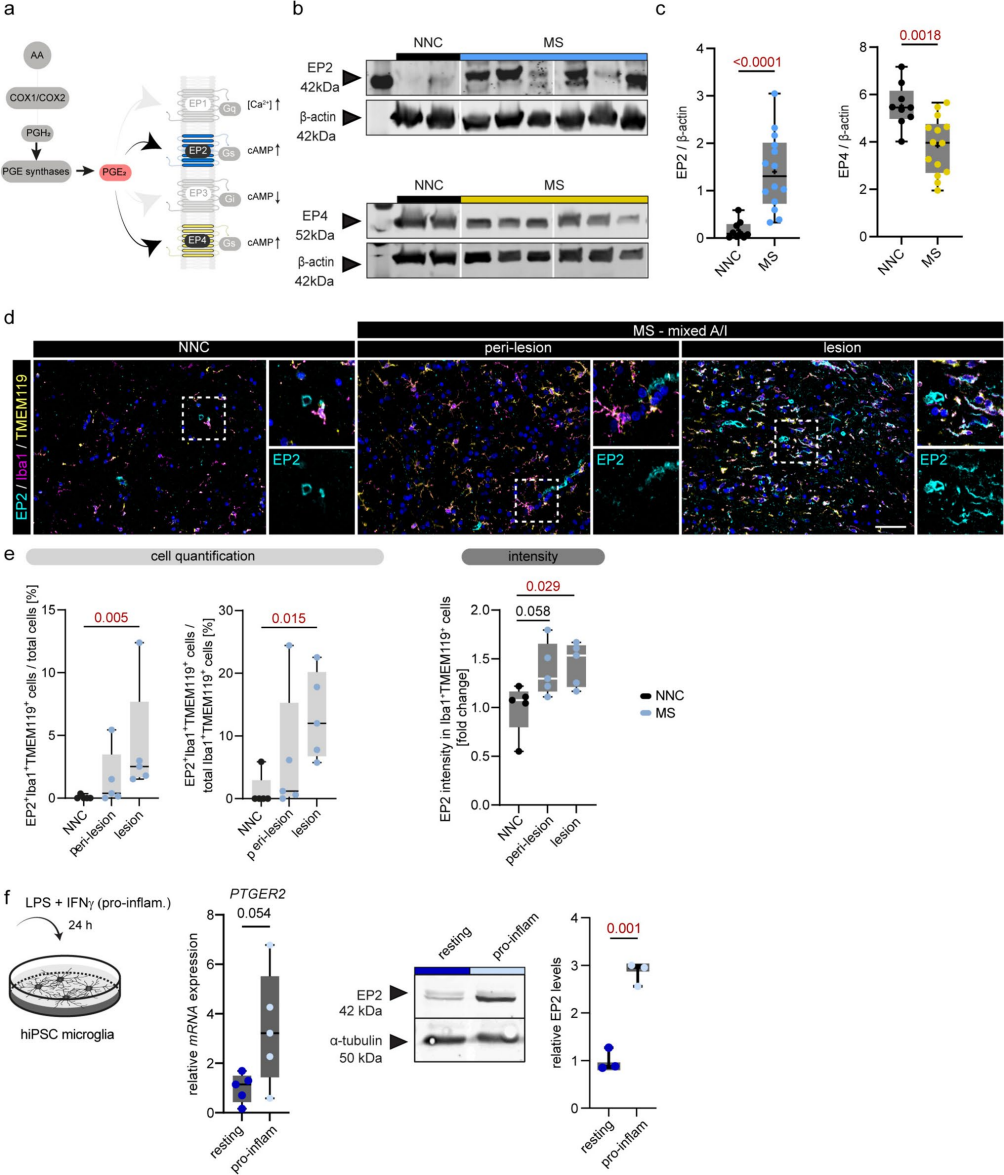
Increased microglial EP2 expression in MS lesions. **a** Schematic overview of PGE_2_ receptors EP1-4 and their G-protein coupled signaling pathways. EP2 (blue) and EP4 (yellow) are highlighted. **b** Representative cropped immunoblots of EP2, EP4 (upper panel each), and β-actin (lower panel) from human brain tissue homogenates of NNC and MS lesions. **c** Densitometric quantification of EP2 and EP4, normalized to β-actin in NNC (*N* = 9) and MS lesions (*N* = 14).** d** Representative images of EP2 (cyan), Iba1 (magenta) and TMEM119 (yellow) immunoreactivity in WM of NNC and mixed A/I MS lesions (peri-lesion and lesion). Panels show outlined excerpts at higher magnification; scale bar: 50 µm. **e** Quantification of EP2^+^ cells and EP2 mean fluorescent intensity within microglia (Iba1^+^TMEM119^+^ cells) in NNC and MS tissues (*N* = 5). **f** *mRNA* expression of *PTGER2* (encoding EP2) and EP2 protein were measured in human iPSC-derived microglia (hiPSC microglia) non-stimulated (resting) or stimulated with LPS + IFNγ for 24 h (pro-inflam). Data is shown as box plots with median ± quartiles; whiskers extend to minimum and maximum. Data have been statistically tested for three groups by ordinary one-way ANOVA with Dunnett’s correction or Kruskal–Wallis test for non-normally distributed data and Dunn’s post hoc analysis. For two groups, an unpaired student t-test with Welch’s correction was used when the variance of the groups was significantly different or the Mann–Whitney test for non-parametric datasets. Exact p-values are reported and statistical significance is set at *p* < 0.05 (red)

PGE_2_ signaling following its local production occurs through four GPCR receptors (EP1-4), of which EP2 and EP4 have been previously related to neurological diseases and neuroinflammation (Fig. 4a) [2, 30]. To investigate the putative local PGE_2_-induced signaling pathway in MS tissues, we next analyzed protein levels of EP2 and EP4 in human brain tissue homogenates of NNCs and MS lesions. Receptor levels of EP4 were significantly lower in MS lesions (*p* = 0.0018) compared to NNCs, whereas EP2 levels were increased in MS lesions compared to NNCs (*p* < 0.0001) (Fig. 4b, c). IHC analysis revealed a co-localization of EP2 with microglia (defined as Iba1^+^TMEM119^+^ cells), and to a far lesser extent with the vasculature (Fig. 4d). The percentage of EP2^+^ microglia of all cells as well as the percentage of EP2^+^ microglia from all Iba1^+^TMEM119^+^ cells was significantly higher in the lesion (mixed A/I) compared to NNC (*p* = 0.005; *p* = 0.015). Microglial EP2 expression (MI) was higher in the lesion (*p* = 0.029) and showed a positive trend in the peri-lesional tissue (*p* = 0.058) compared to NNC (Fig. 4e). Together, MS WM lesions show a transition towards overall decreased EP4 receptor expression and an enhanced EP2 receptor expression, the latter being specifically expressed by microglia.

To mimic microglial PGE2 signaling in MS lesions in vitro, we cultured human iPSC-derived microglia (hiPSC microglia) under resting and pro-inflammatory conditions (LPS/IFNγ for 24 h). Pro-inflammatory microglia showed higher expression of *PTGER2*/EP2 on RNA and protein levels (*p* = 0.054; *p* = 0.001, respectively) when compared to the non-stimulated control (resting) (Fig. 4f). *PTGER4*/EP4 showed an upward trend in pro-inflammatory microglia compared to resting but no clear increase on the protein level (Fig. S3a, b), together suggesting that EP2 signaling might play a more dominant role under inflammatory conditions.

### EP2 signaling regulates both PGE_2_-related activation and resolution of microglia

**Fig. 5:**
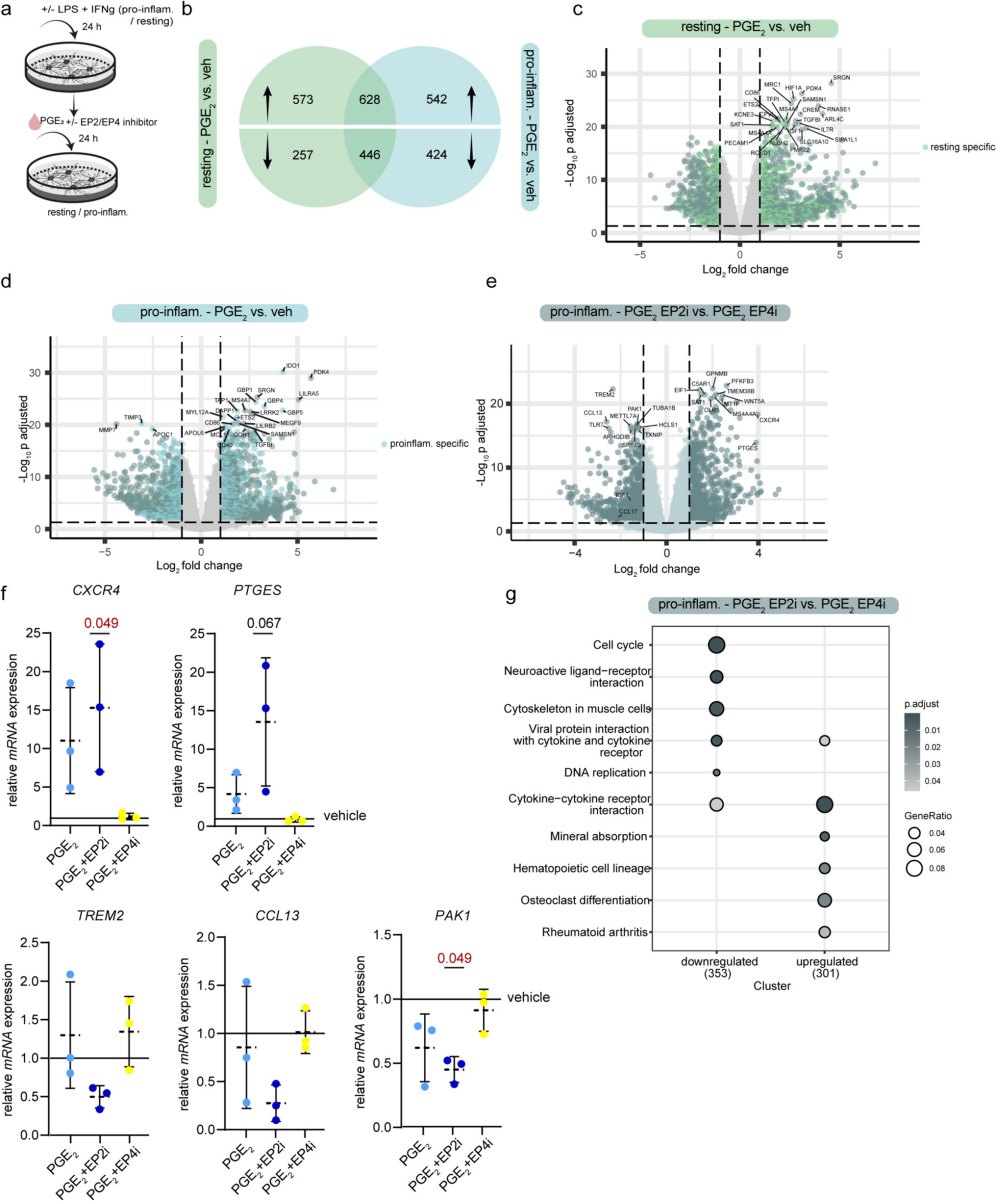
PGE_2_ signaling adds to immunological response in microglia. **a** Schematic overview of PGE_2_ and EP inhibitor treatment strategy on resting and pro-inflammatory hiPSC microglia. **b** Venn diagram of significantly differentially expressed genes (DEG) presenting upregulated (top) and downregulated genes (bottom) in resting (green) vs. pro-inflammatory (blue) microglia treated with PGE_2_ vs. vehicle; *N*_TR_ = 5. **c** Volcano plot representing significant DEGs comparing PGE_2_ treatment versus vehicle in resting microglia with respect to -log_10_ p adjusted in the *y*-axis and log_2_ fold change in the *x*-axis. **d** Volcano plot representing significant DEGs comparing PGE_2_ treatment vs. vehicle in pro-inflammatory microglia. **e** Volcano plot visualizing DEGs regulated by EP2i + PGE_2_ vs EP4i + PGE_2_ in pro-inflammatory microglia. **f** mRNA expression of *CXCR4*, *PTGES*, *TREM2, CCL13,* and *PAK1* in pro-inflammatory microglia treated with PGE_2_ + EP inhibitors; *N* = 3. **g** Over-representation analysis of enriched KEGG pathways in pro-inflammatory iPSC microglia treated with PGE_2_ + EP2i vs. PGE_2_ + EP4i. Data is shown as box plots with median ± quartiles; whiskers extend to minimum and maximum. Data have been statistically tested for four groups by Friedman test for non-normally distributed data and Dunn’s post hoc analysis. Exact p-values are reported and statistical significance is set at *p* < 0.05 (red)

To study the consequences of PGE_2_ signaling in microglia in-depth, we next treated both resting and pro-inflammatory hiPSC microglia with PGE_2_ (1 µM) or vehicle (0.1% ethanol) in combination with a selective EP2 or EP4 inhibitor (EP2i/EP4i) (Fig. 5a). Using bulk RNA sequencing, we identified a total of 12,111 genes and PGE_2_ treatment induced upregulation of 1201 genes in resting microglia and 1170 in pro-inflammatory microglia respectively, of which 628 genes were shared between the conditions (Fig. 5b). 703 genes were downregulated in resting and 870 in pro-inflammatory microglia, with 446 genes shared between resting and pro-inflammatory microglia (Fig. 5b) (Table S1). PGE_2_ addition to resting microglia induced differentially expressed genes (DEGs), including immunity-related genes (*CD86, MRC-1 (CD206)* and *IL7R*), ischemia-modulated genes (*HIF1A*, *SRGN*) and transcription-related genes (*RNAseI, ETS-2, CREM*) (Fig. 5c) (Table S1). Interestingly, EP2 but not EP4 inhibitors had a drastic effect on PGE_2_-treated resting microglia (Fig. S4a). Of note, PGE_2_ addition to pro-inflammatory microglia also enhanced the expression of immunity-related genes, including *CD86, CD40*, and members of the leukocyte immunoglobulin-like receptor (LIR) family (Fig. 5d) (Table S1). In addition, guanylate-binding proteins (GBP) family members 1,4,5, which play a central role in protective immunity against pathogens are upregulated by PGE_2_ (Fig. 5d)_._ In line with this, PGE_2_ addition downregulated osteopontin (*SPP1*), matrix metalloprotease 7 (*MMP7*) and 24-dehydrocholesterol reductase (*DHCR24*) expression, indicating initiation of a pro-resolving phagocytic phenotype (Fig. 5d) (Table S1).

Both EP2i and EP4i conditions separate clearly in pro-inflammatory microglia from the PGE_2_ and vehicle conditions (Fig. S4b). DEGs between PGE_2_ + EP2i treatment compared to PGE_2_ + EP4i in pro-inflammatory microglia are visualized in the volcano plot (Fig. 5e) (Table S2). Compared to EP2i-treated cells, EP4i treatment induced expression of homeostatic/pro-resolving markers (*IGF1, TREM2*), cytokine signaling (*CCL13*), nuclear signaling and cytoskeleton rearrangement (*PAK1, TUBA1B, HCLS1, SRSF3*) genes (Fig. 5e) (Table S2). EP2i promoted higher expression of genes related to chemokine-receptor signaling (*cxcr4*) as well as PGE_2_ synthase (*PTGES*) compared to EP4i (Fig. 5e) (Table S2). Validation experiments in pro-inflammatory microglia showed EP2i enhanced PGE_2_-induced *CXCR4* (*p* = 0.049) and *PTGES* (*p* = 0.067) mRNA expression (Fig. 5f). EP2i further reduced *PAK1* (*p* = 0.049), *CCL13*, and *TREM2* expressions*;* however, the latter two did not reach significance (Fig. 5f). Of note, comparing the EP2^+^ versus EP2^−^ cells within the Iba1^+^ population in MS lesion IHC revealed an increased intensity of the homeostatic microglia marker TMEM119 in EP2^+^ cells (*p* = 0.0699) (Fig. S5). In pro-inflammatory microglia, pathways over-representation analysis (KEGG pathways) showed a lower expression of genes associated with cell cycle pathways and some cytokine–receptor interactions when comparing EP2i to EP4i, but also a higher expression of genes in this pathway category (Fig. 5g) (Table S3). Taken together, these findings suggest that PGE_2_ might stimulate an inflammatory response in homeostatic microglia, but that it also guides activated microglia towards inflammation resolution. A clear differentiation between EP2 and EP4 signaling is challenging but our findings suggest that PGE_2_-EP2 signaling could be responsible for a more resolving signature under inflammatory conditions.

## Discussion

Chronic neuroinflammation in the CNS of PwMS presents a major challenge for therapeutic intervention, routed in the lack of understanding the underlying dysregulated inflammatory/resolving pathways. In our study, we depict a distribution map of the lipids AA and PGE_2_ in WM MS brain lesions and highlight the role of PGE_2_-EP2 signaling in activated microglia at the lesion site. Specifically, we show a reduction of AA in MS brain tissues compared to NNCs and a distinct loss within MS lesions compared to surrounding tissue. *PTGS2* and *PTGES* expression increased in MS tissue lysates and we report a higher PGE_2_/AA ratio in demyelinated tissue areas in MS. Within the same tissue location, PGE_2_ receptor EP2 was upregulated in microglia, resulting in overall higher levels of EP2 in MS lesions compared to NNCs, whereas EP4 decreased in MS lesions. Finally, we focus on the genes modulated by PGE_2_ signaling in resting and activated microglia and investigate specific pathways evoked through PGE_2_-EP2 and/or PGE_2_-EP4 signaling.

Lipid brain constituents assessed by lipidomics on brain tissue homogenates is a useful tool for defining pathology-induced metabolic differences; however, it lacks the spatial element to pinpoint lipid changes relative to its environment [36]. Here, we used MSI on FF human WM brain tissue slides from NNC and PwMS to explore spatial lipid distribution within the CNS. With this technique, we created a precise map of AA and PGE_2_ abundances in and around MS lesions and linked them to pathological hallmarks of MS such as demyelination and immune cell activation. We found that AA concentrations were significantly reduced in MS tissues compared to NNCs and for both AA and PGE_2_, we observed very distinct patterns of alterations in and around the MS lesions. In a recent mouse study, the lipid profile of lysolecithin-induced focal demyelinated lesion homogenates was tracked [57]. 14 days after injection, free AA and PGE_2_ levels increased. As AA release from the membrane is stimulated by inflammation, free AA levels could initially increase (as seen after 14 days in the animal model) but later decrease due to an increased AA conversion into downstream bioactive LMs such as prostaglandins and leukotrienes [22]. Data on other AA-derived LMs such as hydroxy-eicosatetraenoic acids and leukotrienes in our data set further support this hypothesis (data not shown). Likely, reduced storage and enhanced metabolism synergistically create the overall and lesion-specific AA reduction observed in MS. Importantly, the cases used in our study do not equally represent both sexes, particularly in the MS cohort of the MSI experiments, which we acknowledge as a limitation of the study.

In MS tissues, we observed an increase of prostaglandin-converting enzymes *PTGS2* (COX2) and *PTGES* (mPGES-1) mRNA expression which further supports the hypothesis of locally increased PGE_2_ biosynthesis. Among the three prostaglandin isozymes, mPGES-1 is functionally coupled to COX2 and increases concomitantly with COX2 in response to various stimuli [68]. For example, COX2 and m-PGES-1 are induced in the MS mouse model experimental autoimmune encephalomyelitis (EAE) and in MS lesions [34]. Further, we report COX2 expression in the vasculature and perinuclear in CD45^+^ cells but observed it less in the cytoplasm. These observations are in line with previous reports in primary human cells [43, 56]. The latter observation confirmed other studies on COX2 in macrophages and microglia of MS lesions, particularly in the process of demyelination [66, 83]. Of note, despite increased mRNA expression of *PTGS2* (COX2) in MS, we did not observe overall higher levels of COX2 immunoreactivity in our MS tissues compared to NNCs and also found COX2^+^ cells in our control tissues counter to a previous study [66]. Local heightened COX2 levels upon active lipid metabolism may stay undetected in the general comparison taken and are, therefore, still plausible.

The source of PGE_2_ is proposed to be a production by inflamed endothelial cells, dying oligodendrocytes, activated microglia/macrophages as well as direct transport from the blood [25, 38, 50]. Our ion images show that PGE_2_ /AA ratios are highest in demyelinated lesion areas where axonal damage and microglia activation take place [3, 33]. Similarly, CNS PGE_2_ detected concentration was found to be increased at the peak of the disease in EAE and cuprizone-fed mice, rodent models for MS representing the inflammatory and demyelinating aspects respectively [34, 55]. Inhibition of PGE_2_ production by COX2 inhibition in EAE and COX2 KO mice in the cuprizone model showed reduced clinical scores and less oligodendrocyte death [10, 16]. Despite positive results in mouse models, COX2 inhibition in humans is connected to increased risk for cardiovascular events and (hemorrhagic) stroke [47, 75]. Consequently, a more specific target, such as one/multiple of the four PGE_2_ receptors, would be desirable to largely circumvent the impairment of crucial cellular functions for body homeostasis.

PGE_2_ exerts beneficial and detrimental immunomodulatory functions by signaling through EP2 and EP4; however, the specific pathways initiated in the CNS of PwMS remain unclear. Our data show a decrease in EP4 expression in MS lesions, which contrasts with findings in EAE mouse models where EP4 is upregulated in spinal cord lysates and blood [34, 69]. However, EP4 expression is also reduced in blood cells from MS patients [58]. Previous studies have indicated species-specific differences, with humans exhibiting higher EP4 expression in microglia compared to mice [24]. These discrepancies suggest that EP4 regulation may vary between species and is influenced by the pathological context (MS versus EAE). In our study, we report a significant increase of EP2 in the homogenates of MS tissues compared to NNCs. EP2 expression was predominantly observed in microglia at the MS lesion site, which together with the increased PGE_2_/AA ratio at this site, suggests enhanced PGE_2_-EP2 signaling in MS lesions. Induced EP2 and EP4 expression have been shown in vivo in the CNS of EAE and cuprizone mouse models, where EP2 increases prior to EP4 upregulation [15, 34, 55]. Previous studies revealed that HIF-1a, induced by high cell density, downregulates EP4 expression, but not EP2 [54, 70]. As HIF-1a is also expressed in demyelinating WM MS lesions, it may regulate the reduced EP4 expression in MS compared to NNCs [1, 18]. In addition to the shift of expression, EP2 receptors are to a lesser extent desensitized by PGE_2_ binding than EP4, plus EP2 also respond (gradually less) to the downstream metabolite 15-keto-PGE_2_ further strengthening the hypothesis of enhanced PGE_2_-EP2 signaling [51]. Importantly, we were not able to assess the cell-specific expression of EP4 in our brain tissue due to a lack of appropriate EP4 antibodies; hence, we can only make conclusions on its expression relative to NNC tissues but not cell-specific. In conclusion, our findings suggest enhanced PGE_2_–EP2 signaling in MS compared to NNC, mostly restricted to inflamed microglia in MS lesions.

Inhibiting EP2 or EP4 activity has been promising in diverse disease models for inflammation [14], demyelination [56], cognition [48], epileptic seizures [49], and Alzheimer’s disease pathology [23]. Specifically, during EAE, a T-cell-driven MS disease mouse model, EP4 inhibition during the immunization phase was effective in reducing the clinical score, while EP2-deficient mice did not show any beneficial effect unless used together with an EP4 antagonist [14]. On the contrary, EP2 antagonism was effective in reducing demyelination in a cuprizone model by preventing oligodendrocyte apoptosis and in LPS-injected (i.p.) mice by reducing microgliosis [28, 56]. Hence, the development of a safe BBB-penetrating EP2 antagonist for clinical exploration is ongoing, and EP4 antagonists currently undergo clinical trials for cancer immunotherapy [17, 26, 44]. In light of our observed enhanced PGE_2_–EP2 signaling in MS, EP2 targeting might be the most interesting therapeutic candidate; however, effects are likely highly dependent on cell state and tissue microenvironment.

Focusing on the specific effect in the CNS, we lastly aimed to shed light on the bimodal nature of PGE_2_ signaling in both resting and activated microglia using RNA sequencing. Primarily, we found that PGE_2_ treatment polarizes resting microglia toward an inflammatory response and guides activated microglia toward resolution and a regulated immune response. Inhibiting EP2 signaling in resting microglia suppressed part of the PGE_2_-induced inflammation signature, whereas EP4 inhibition was less effective. Pro-inflammatory effects initiated by LPS/IFNγ treatment were dampened both through PGE_2_-EP2, but mainly PGE_2_-EP4 inhibition. Of note, EP2 blockage in activated microglia resulted in a trend towards downregulation of *TREM2*, a key player in the maintenance of microglia metabolism and response to phagocytic triggers, and an upregulation of *PTGES*, potentially exacerbating further PGE_2_ synthesis [72]. Contextualizing these findings to demyelinating lesion tissue of MS patients, in which we found increased microglial EP2 expression in the lesion and higher TMEM119 intensity in EP2^+^ cells, the enhanced EP2 signaling could be a response mechanism to counteract demyelination and induce a potential resolving microglial phenotype. Thus, although the underlying signaling remains to be validated, PGE_2_-EP2 signaling might also have crucial beneficial effects in MS lesions.

To conclude, in the current research, we provide a spatial distribution of AA and its derivative PGE_2_ in WM MS lesions, which we coupled to enhanced PGE_2_-EP2 signaling. This could potentially be a chronic resolution response dampening neuroinflammation, counteracting the primary pro-inflammatory effects of PGE_2_ in MS.

## Supplementary Information

Below is the link to the electronic supplementary material.

Supplementary file1 (XLSX 464 kb)

Supplementary file2 (XLSX 154 kb)

Supplementary file3 (XLSX 10 kb)

Supplementary file4 (DOCX 37 kb)

Supplementary file5 (TIF 6774 kb)

Supplementary file6 (DOCX 386 kb)

Supplementary file7 (DOCX 28 kb)

Supplementary file8 (DOCX 25 kb)

Supplementary file9 (TIF 13114 kb)

Supplementary file10 (TIF 518 kb)

Supplementary file11 (TIF 3477 kb)

Supplementary file12 (TIF 1692 kb)

Supplementary file13 (TIF 1745 kb)

## Data Availability

All data presented in this study are available from the corresponding author upon reasonable request.

## Abbreviations

AA: Arachidonic acid
BBB: Blood–brain barrier
BMEC: Brain microvascular endothelial cell
CBF: Cerebral blood flow
CCL13: C-C motif chemokine ligand 13
CNS: Central nervous system
COX: Cyclooxygenase
CSF: Cerebrospinal fluid
CXCR4: C-X-C motif chemokine receptor 4
DHCR24: 24-Dehydrocholesterol reductase
EAE: Experimental autoimmune encephalomyelitis
EP2: Prostaglandin E2 receptor 2
EP4: Prostaglandin E2 receptor 4
FF: Fresh frozen
FFPE: Formalin-fixed paraffin-embedded
GBP: Guanylate-binding proteins
GPCR: G-protein coupled receptor
LIR: Leukocyte immunoglobulin-like receptor
LM: Lipid mediator
MMP7: Matrix metalloprotease 7
MS: Multiple sclerosis
MSI: Mass spectrometry imaging
NAWM: Normal-appearing white matter
NNC: Non-neurological control
PGE_2_: Prostaglandin E2
PMS: Progressive MS
PPMS: Primary progressive MS
PUFA: Polyunsaturated fatty acid
PwMS: People with MS
SPMS: Secondary progressive MS
TREM2: Triggering receptor expressed on myeloid cells 2
UEA I: Ulex Europaeus Agglutinin I
WM: White matter
