# Supplementary material for: Spatial mapping of the AA-PGE2-EP axis in multiple sclerosis lesions
Source: Acta Neuropathol. 2025 Apr 29;149(1):39. doi: 10.1007/s00401-025-02878-3 (PMC12041062; doi:10.1007/s00401-025-02878-3)
Supplement: Supplementary file 4 — Supplementary file4 (DOCX 37 kb) [file 401_2025_2878_MOESM4_ESM.docx]

Table 1: Clinical and demographic data of MS and NNC subjects organized by experiments

| **Case ID** | **Age at death** | **Sex** | **PMD (h)** | **Cause of death** | **Type of MS** | | **Tissue preservation** | | **Lesion type** | **MSI** | | **mRNA** | | **IHC COX2** | | **WB**  **EP2/4** | | | **IHC EP2** |
| --- | --- | --- | --- | --- | --- | --- | --- | --- | --- | --- | --- | --- | --- | --- | --- | --- | --- | --- | --- |
| NNC019 | 68 | M | 08:40 | Euthanasia | n/a | | FF, FFPE | | n/a | y | | y | | y | |  | | | y |
| NNC945 | 59 | F | 08:10 | Euthanasia, terminal COPD | n/a | | FF | | n/a | y | | y | | y | |  | | | y |
| NNC989 | 71 | F | 07:50 | Lung carcinoma | n/a | | FF | | n/a | y | | y | | y | |  | | | y |
| MS16 | 77 | F | 09:45 | Aspiration pneumonia | SPMS | | FF, FFPE | | A/I | y | | y | | y | |  | | | y |
| MS46 | 51 | F | 09:10 | Euthanasia | SPMS | | FF, FFPE | | A | y | | y | | y | |  | | | y |
| MS68 | 48 | F | 09:20 | Pneumonia | unk | | FF | | AI/I | y | | y | | y | |  | | |  |
| MS60 | 60 | F | 05:05 | Euthanasia | SPMS | | FF | | A/I | y | | y | | y | |  | | |  |
| MS42 | 82 | F | 07:30 | Cardiac arrest, sudden death | PPMS | | FF | | A/I | y | | y | | y | |  | | |  |
| MS32 | 54 | M | unk | Progressive dyspneu | unk | | FF | | A | y | | y | | y | |  | | |  |
| MS116 | 66 | F | 09:30 | Euthanasia | SPMS | | FF | | CIA | y | | y | | y | |  | | |  |
| NNC999 | 74 | M | 10:20 | Euthanasia | n/a | | FF | | n/a |  | | y | | y | |  | | |  |
| NNC227 | 93 | M | 06:00 | Cachexia, cerebrovascular accident/kidney failure | n/a | | FF | | n/a |  | | y | |  | |  | | |  |
| NNC254 | 75 | F | 05:40 | Cachexia | n/a | | FF | | n/a |  | | y | |  | |  | | |  |
| NNC275 | 82 | F | 05:10 | Pneumonia by haemothorax | n/a | | FF | | n/a |  | | y | |  | |  | | |  |
| NNC215 | 79 | M | 09:00 | Pneumonia and metastasized kidney carcinoma | n/a | | FF | | n/a |  | | y | |  | |  | | |  |
| MS120 | 73 | M | 08:45 | Urosepsis | SPMS | | FF | | A/I |  | | y | |  | |  | | |  |
| MS100 | 71 | F | 07:05 | Cachexia with PMS and metastatic breast cancer | PMS | | FF | | A/I |  | | y | |  | |  | | |  |
| MS298 | 53 | F | 10:45 | Euthanasia | PMS? | | FF | | unk |  | | y | |  | |  | | |  |
| MS51 | 47 | M | 07:15 | Urosepsis with organ failure | SPMS | | FF | | unk |  | | y | |  | |  | | |  |
| MS139 | 56 | M | 08:00 | Pneumonia | SPMS | | FF | | unk |  | | y | |  | |  | | |  |
| NNC1 | 41 | F | 13:30 | Pulmonary hemorrhage | n/a | | FF | | n/a |  | |  | |  | | y | | |  |
| NNC2 | 98 | M | 08:40 | Aortic dissection | n/a | | FF | | n/a |  | |  | |  | | y | | |  |
| NNC3 | 82 | M | 13:35 | Heart failure | n/a | | FF | | n/a |  | |  | |  | | y | | |  |
| NNC4 | 53 | M | 14:25 | Cardiac complications | n/a | | FF | | n/a |  | |  | |  | | y | | |  |
| NNC5 | 78 | M | 05:55 | Cardiac failure | n/a | | FF | | n/a |  | |  | |  | | y | | |  |
| NNC6 | 77 | F | 09:15 | Sudden death | n/a | | FF | | n/a |  | |  | |  | | y | | |  |
| NNC7 | 88 | F | 05:40 | Cardiac failure | n/a | | FF | | n/a |  | |  | |  | | y | | |  |
| NNC8 | 77 | F | 19:45 | Malignant lymphoma | n/a | | FF | | n/a |  | |  | |  | | y | | |  |
| NNC9 | 91 | F | 05:45 | Sudden death | n/a | | FF | | n/a |  | |  | |  | | y | | |  |
| MS1 | 70 | F | 06:30 | Probably urosepsis | SPMS | | FF | | unk |  | |  | |  | | y | | |  |
| MS2 | 53 | F | 10:45 | Euthanasia | SPMS | | FF | | unk |  | |  | |  | | y | | |  |
| MS3 | 48 | F | 04:50 | Euthanasia | SPMS | | FF | | unk |  | |  | |  | | y | | |  |
| MS4 | 48 | F | 08:10 | Euthanasia | SPMS | | FF | | unk |  | |  | |  | | y | | |  |
| MS5 | 75 | F | 08:00 | Pneumonia | SPMS | | FF | | unk |  | |  | |  | | y | | |  |
| MS6 | 43 | M | 08:30 | Pneumonia | SPMS | | FF | | unk |  | |  | |  | | y | | |  |
| MS7 | 53 | M | 05:53 | Pneumonia | PPMS | | FF | | unk |  | |  | |  | | y | | |  |
| MS8 | 52 | F | 08:25 | Pneumonia | PMS | | FF | | unk |  | |  | |  | | y | | |  |
| MS9 | 48 | F | 05:50 | Congestive cardiac failure | SPMS | | FF | | unk |  | |  | |  | | y | | |  |
| MS10 | 66 | F | 06:20 | Cancer | SPMS | | FF | | unk |  | |  | |  | | y | | |  |
| MS11 | 71 | F | 10:15 | Post-surgery respiration problems | PMS | | FF | | unk |  | |  | |  | | y | | |  |
| MS12 | 64 | M | 07:30 | End stage prog. MS | PPMS | | FF | | unk |  | |  | |  | | y | | |  |
| MS13 | 65 | M | 10:35 | Urosepsis | SPMS | | FF | | unk |  | |  | |  | | y | | |  |
| MS14 | 77 | M | 04:15 | Cerebral vascular accident | PPMS | | FF | | unk |  | |  | |  | | y | | |  |
| NNC095 | 68 | M | 07:35 | Esophageal carcinoma, narrowing aa. coronariae | n/a | | FFPE | | n/a |  | |  | |  | |  | | | y |
| NNC880 | 77 | M | 11:25 | Pneumonia | n/a | | FFPE | | n/a |  | |  | |  | |  | | | y |
| MS71 | 67 | M | 07:55 | Euthanasia | unk | | FFPE | | A/I |  | |  | |  | |  | | | y |
| MS85 | 67 | F | 11:25 | Pneumonia | unk | | FFPE | | A/I |  | |  | |  | |  | | | y |
| MS31 | 53 | F | 05:50 | Euthanasia | PMS | | FFPE | | A/I |  | |  | |  | |  | | | y |
|  | | | | | | MSI | | | qPCR | | COX2 | | WB | | | | EP2 IHC | | |
| Female (%) | | | | | | C | | M | C | M | C | M | C | | M | | C | M | |
|  |  |  |  |  |  | 66 | | 86 | 50 | 66 | 50 | 86 | 56 | | 66 | | 40 | 80 | |

^PMD Post-mortem delay, unk unknown, n/a not applicable, mRNA messenger RNA, PMS progressive MS, PPMS primary progressive MS, SPMS secondary progressive MS, FF fresh frozen, FFPE formalin-fixed paraffin-embedded, CIA chronic inactive, A/I mixed active/inactive, A active, MSI Mass Spectrometry Imaging, C control, M MS^
