# Supplementary figures and images for: Spatial mapping of the AA-PGE2-EP axis in multiple sclerosis lesions
Source: Acta Neuropathol. 2025 Apr 29;149(1):39. doi: 10.1007/s00401-025-02878-3 (PMC12041062; doi:10.1007/s00401-025-02878-3)

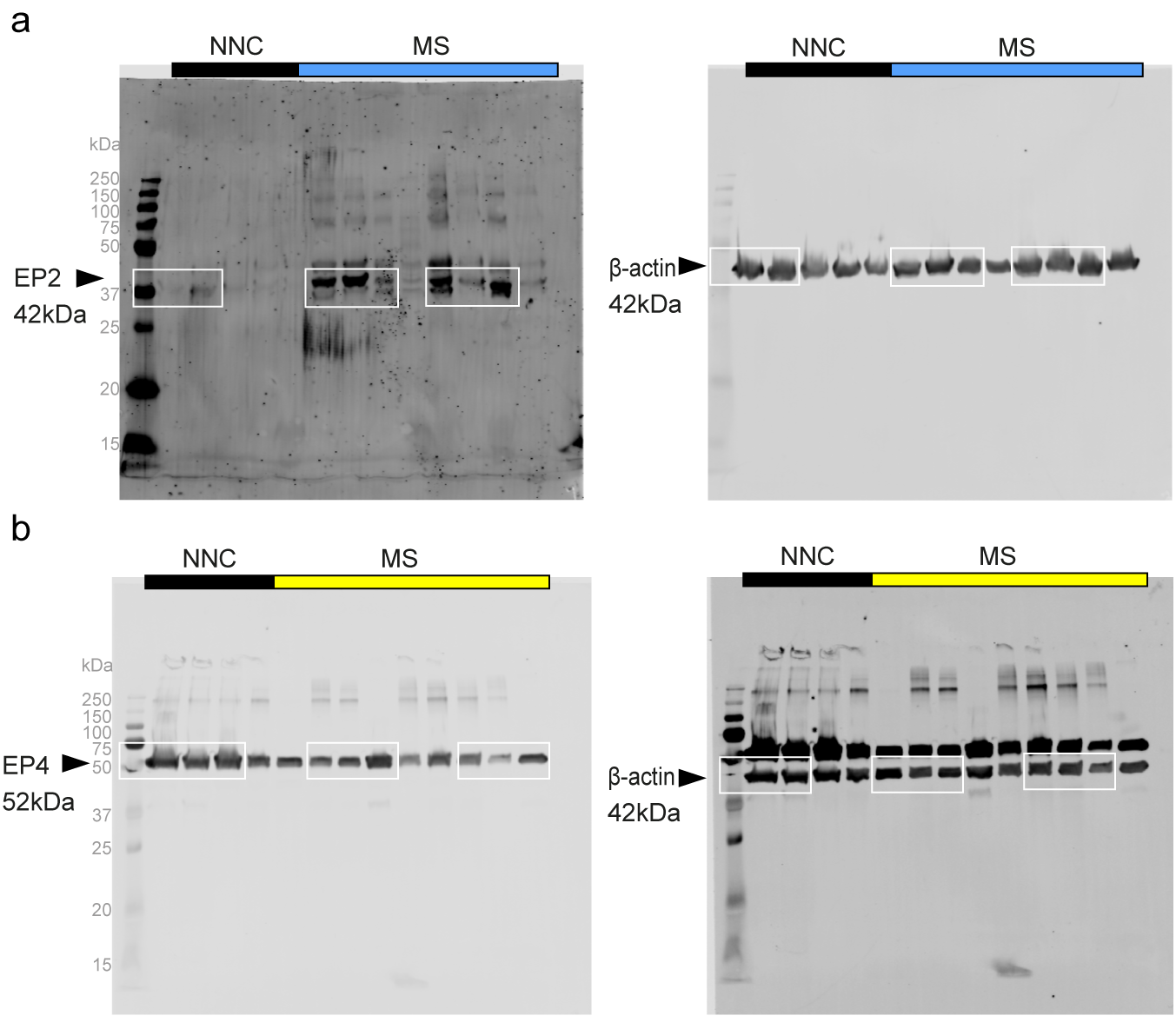

Supplement: Supplementary file 5 — Supplementary file5 (TIF 6774 kb) [file 401_2025_2878_MOESM5_ESM.tif]

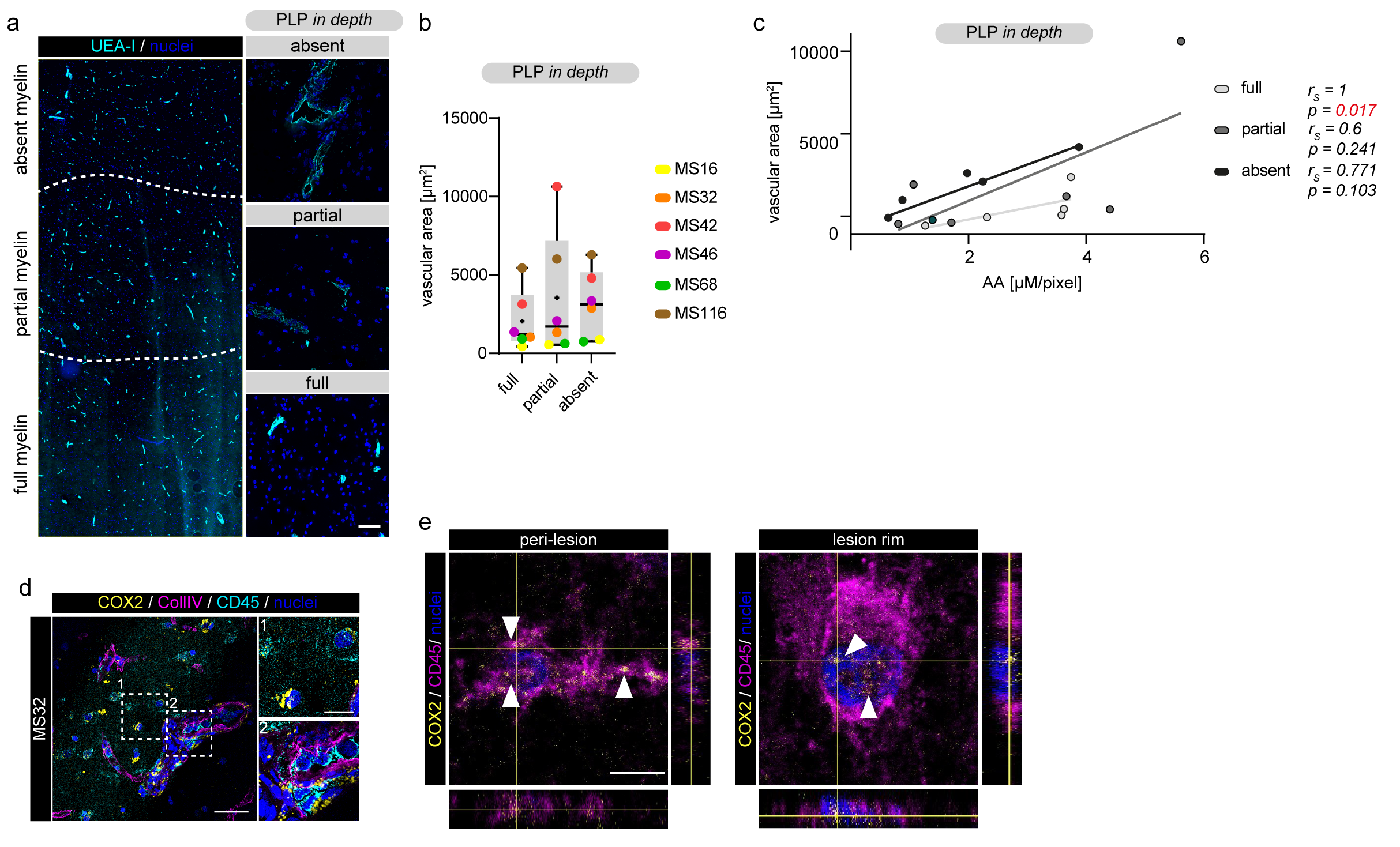

Supplement: Supplementary file 9 — Supplementary file9 (TIF 13114 kb) [file 401_2025_2878_MOESM9_ESM.tif]

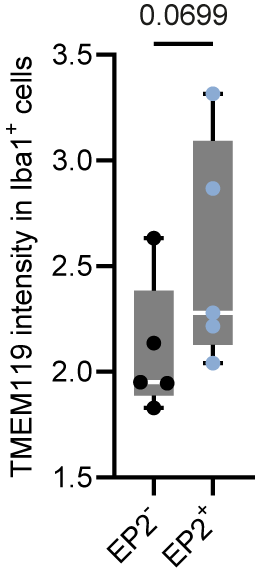

Supplement: Supplementary file 10 — Supplementary file10 (TIF 518 kb) [file 401_2025_2878_MOESM10_ESM.tif]

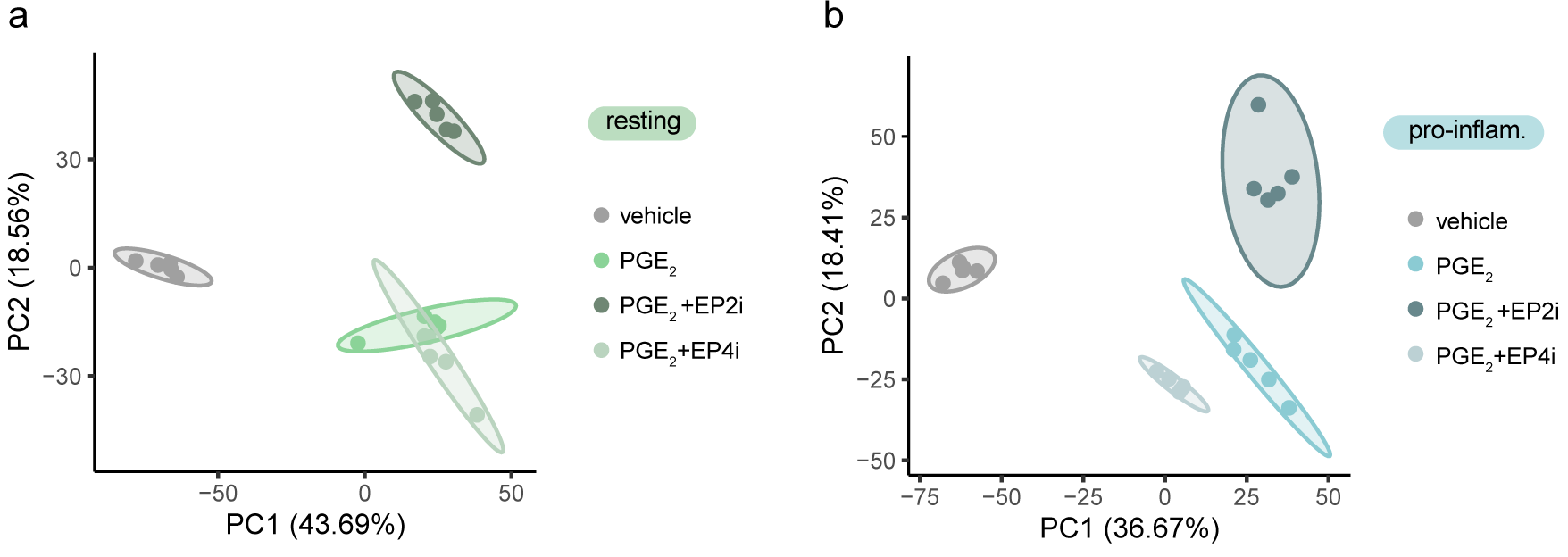

Supplement: Supplementary file 11 — Supplementary file11 (TIF 3477 kb) [file 401_2025_2878_MOESM11_ESM.tif]

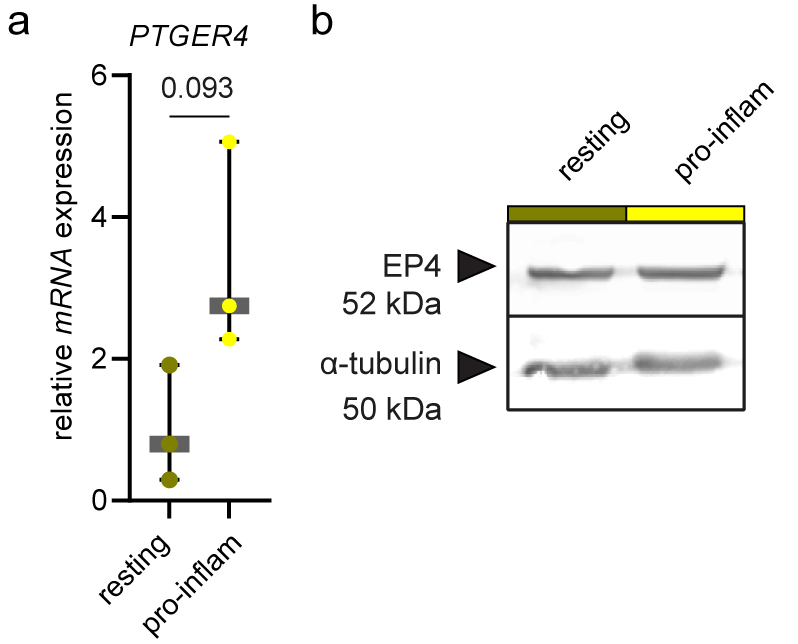

Supplement: Supplementary file 12 — Supplementary file12 (TIF 1692 kb) [file 401_2025_2878_MOESM12_ESM.tif]

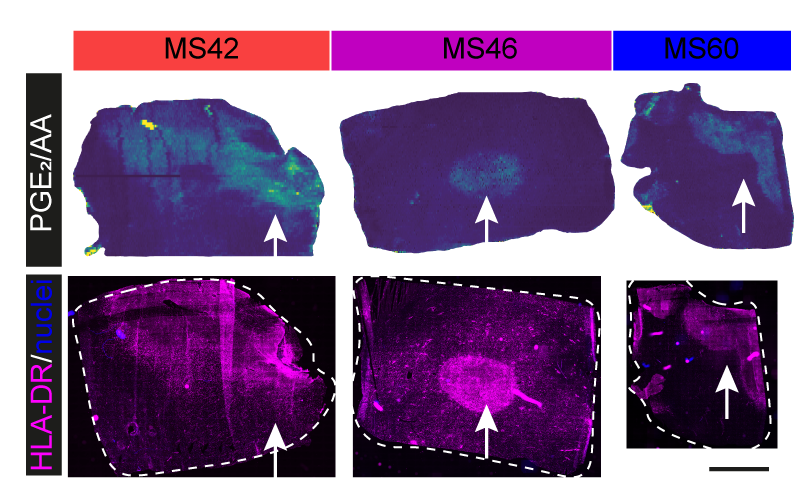

Supplement: Supplementary file 13 — Supplementary file13 (TIF 1745 kb) [file 401_2025_2878_MOESM13_ESM.tif]
