# Supplementary material for: Spatial mapping of the AA-PGE2-EP axis in multiple sclerosis lesions
Source: Acta Neuropathol. 2025 Apr 29;149(1):39. doi: 10.1007/s00401-025-02878-3 (PMC12041062; doi:10.1007/s00401-025-02878-3)
Supplement: Supplementary file 6 — Supplementary file6 (DOCX 386 kb) [file 401_2025_2878_MOESM6_ESM.docx]

**Additional file 1**

**Spatial mapping of the AA-PGE_2_-EP axis in multiple sclerosis lesions**

Cathrin E. Hansen^1,2,3*^, Julia Konings^1,2,3*^, Gabor Toth^5,6^, Serhii Chornyi^1^, Jelle Broos^1,2,3^, Manon Karsten^1^, Bert van het Hof^1^, Susanne M.A. van der Pol^1^, Stephanie D. Beekhuis-Hoekstra^1^, Nine Kok^1^, Wing Ka Fung^1^, Wia Baron^7^, Maarten E. Witte^1,2,3,4^, Ingela Lanekoff^5,6^, Helga E. de Vries^1,2,3^, Gijs Kooij^1,2,3,4#^.

^1^ Amsterdam UMC location Vrije Universiteit Amsterdam, Department of Molecular Cell Biology and Immunology, De Boelelaan 1117, Amsterdam, The Netherlands

^2^ Amsterdam Neuroscience, Amsterdam UMC, Amsterdam, The Netherlands

^3^ MS Center Amsterdam, Amsterdam UMC Location VU Medical Center, Amsterdam, The Netherlands

^4^Amsterdam Institute for Immunology and Infectious Diseases, Amsterdam UMC, Amsterdam, The Netherlands

^5^ Department of Chemistry, BMC, Uppsala University, 75237 Uppsala, Sweden

^6^Center of Excellence for the Chemical Mechanisms of Life, Uppsala University, Sweden

^7^Biomedical Sciences of Cells & Systems, Section Molecular Neurobiology, University of Groningen, UMCG, A. Deusinglaan 1, Groningen, The Netherlands

^*^ Both authors contributed equally to this work

^#^ Corresponding author: Gijs Kooij (email: g.kooij@amsterdamumc.nl, tel: +31 (0) 204448080)

**
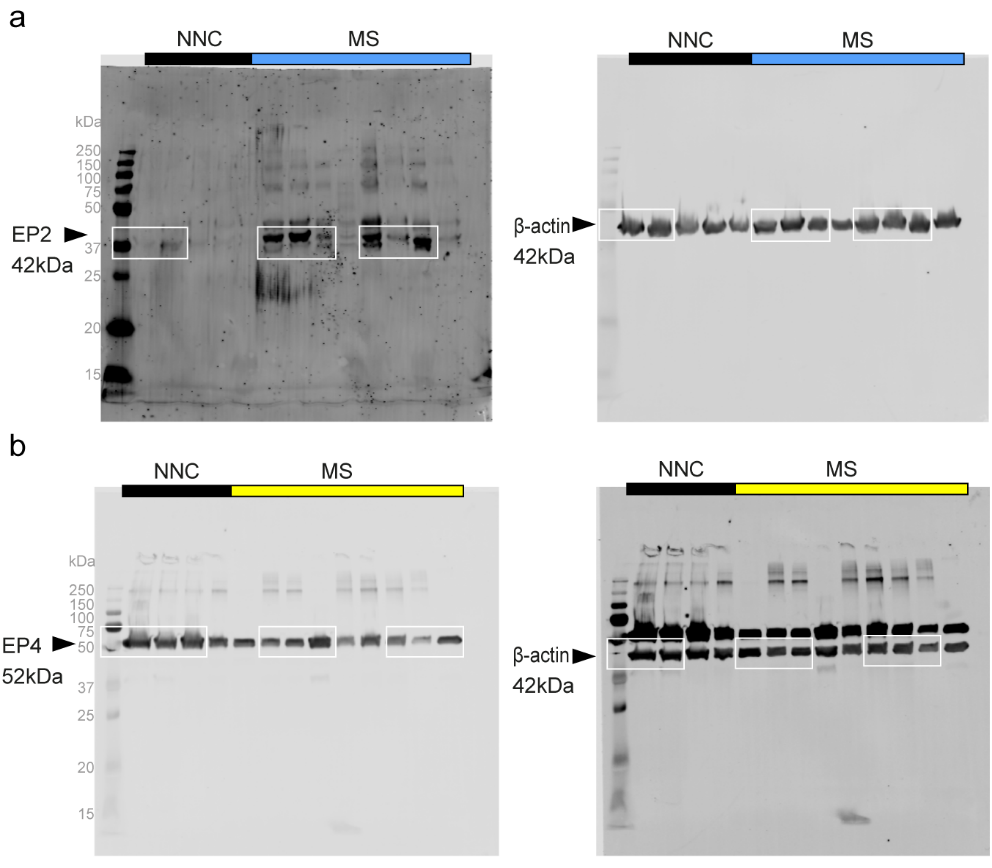
**

**Additional file.1 Original Western blot images a** Immunoblot of EP2 (left) and β-actin (right) on NNC and MS brain tissue lysate (white rectangles indicating representative cropped areas depicted in main figure). **b** Immunoblot of EP4 (left) and β-actin (right) on NNC and MS brain tissue lysates (white rectangles indicating representative cropped areas depicted in main figure).
