# Supplementary material for: Spatial mapping of the AA-PGE2-EP axis in multiple sclerosis lesions
Source: Acta Neuropathol. 2025 Apr 29;149(1):39. doi: 10.1007/s00401-025-02878-3 (PMC12041062; doi:10.1007/s00401-025-02878-3)
Supplement: Supplementary file 7 — Supplementary file7 (DOCX 28 kb) [file 401_2025_2878_MOESM7_ESM.docx]

**Figure legends**

**Spatial mapping of the AA-PGE_2_-EP axis in multiple sclerosis lesions**

Cathrin E. Hansen^1,2,3*^, Julia Konings^1,2,3*^, Gabor Toth^5,6^, Serhii Chornyi^1^, Manon Karsten^1^, Bert van het Hof^1^, Susanne M.A. van der Pol^1^, Stephanie D. Beekhuis-Hoekstra^1^, Nine Kok^1^, Wing Ka Fung^1^, Naomi S. Dijksman^7^, Wia Baron^7^, Maarten E. Witte^1,2,3,4^, Ingela Lanekoff^5,6^, Helga E. de Vries^1,2,3^, Gijs Kooij^1,2,3,4#^.

^1^ Amsterdam UMC location Vrije Universiteit Amsterdam, Department of Molecular Cell Biology and Immunology, De Boelelaan 1117, Amsterdam, The Netherlands

^2^ Amsterdam Neuroscience, Amsterdam UMC, Amsterdam, The Netherlands

^3^ MS Center Amsterdam, Amsterdam UMC Location VU Medical Center, Amsterdam, The Netherlands

^4^Amsterdam Institute for Immunology and Infectious Diseases, Amsterdam UMC, Amsterdam, The Netherlands

^5^ Department of Chemistry, BMC, Uppsala University, 75237 Uppsala, Sweden

^6^Center of Excellence for the Chemical Mechanisms of Life, Uppsala University, Sweden

^7^Biomedical Sciences of Cells & Systems, Section Molecular Neurobiology, University of Groningen, UMCG, A. Deusinglaan 1, Groningen, The Netherlands

^*^ Both authors contributed equally to this work

^#^ Corresponding author: Gijs Kooij (email: g.kooij@amsterdamumc.nl, tel: +31 (0) 204448080)

**Fig.1 Broad and in depth tissue classification of NNC and MS human brain tissue**

**a** Study sampling of three NNCs and seven MS human post-mortem brain tissues. **b** ROIs were defined by differences in neuropathology, assessed by PLP and HLA-DR reactivity. Within the MS tissues, *broad* tissue classes encompass a representative ROI for a lesion, adjacent lesion rim and distant peri-lesional tissue; scale bar: 5 mm**.** For *in depth* quantification, ROIs were stratified by PLP reactivity (A: full, B: partial and C: absent/demyelinated), and HLA-DR density and morphology (1: ramified, low density and inactive lesion; 2: ramified, high density, mixed active/inactive lesion; 3: rounded, ameboid, high density and active lesion); scale bar: 50µm **c** Immunohistochemical depiction of PLP and HLA-DR in MS tissues (color-coded) and annotated ROIs for *broad* and *in depth* tissue classification; scale bar: 5mm.

**Fig.2 Decreased AA levels in WM MS lesions**

**a** Ion images of AA [M + ^107^Ag]^+^ (*m/z* 411.1448 ± 5 ppm) normalized to internal standard AA-d8 in human NNC (n=3) and MS brain tissues (n=7). The spatial distribution of AA is visualized by a min-max intensity scale within each ion image, hence the images are not relative to one another. White arrows point out exemplary demyelinated/lesion areas; red arrows indicate myelinated/peri-lesional tissue. Pixel size is 25 µm x 150 µm; scale bar: 5 mm **b** AA signal intensities were extracted of the whole tissue/donor and normalized to the internal standard of AA-d8 to yield average detected concentration per pixel (µM/pixel). Each dot represents one donor. **c** Paired analysis of AA within MS tissues based on *broad* classification (one representative ROI per tissue area). **d** Paired analysis of AA levels based on *in depth* classification based on PLP or HLA-DR (averages of multiple ROIs per class). **e** *mRNA* expression of various biosynthesizing enzymes in whole tissue block lysates (WBL) of NNC (N= 8) and MS tissues (N= 12). Tissues used both in MSI and qPCR of the MS samples are marked in red. **f** Representative images of COX2, CD45 (immune cell marker) and Collagen IV (Coll IV, vascular marker) immunoreactivity in MS WM tissue; scale bar: 50 µm, zoom in: 25 µm. **g** Quantification of COX2 mean fluorescent intensity (MI) measured within the nuclei and percentage of COX2^+^ cells in NNC (N = 4) and MS lesions (N=6). **h** Paired tissue analysis of COX2^+^ cells in MS tissues (HLA-DR *in depth*) and Spearman correlation (r_s_) of AA levels with COX2^+^ cells (HLA-DR *in depth*). Data is shown as box plots with median ± quartiles; whiskers extend to minimum and maximum. Data have been statistically tested for three groups by Friedman test (paired) for non-normally distributed data and Dunn’s post-hoc analysis. For two groups an unpaired student-t test with Welch’s correction was used when the variance of the groups were significantly different or Mann Whitney test for non-parametric datasets. Exact p-values are reported and statistical significance set at p<0.05 (red).

**Fig.3 PGE_2_/AA levels are increased in demyelinated WM MS tissue**

**a** Ion images of the lipid mediators PGE_2_ (first row) and PGE_2_ /AA ratio images (second row). PGE_2_ ion images were constructed by visualizing m/z 459.1301 ± 5 ppm ([M+^107^Ag]^+^) normalized pixel-by-pixel to internal standard PGE_2_-d9 in human NNC (n=3) and MS lesions (n=7). The spatial distribution of PGE_2_ is visualized by a min-max intensity scale within each single ion image, hence the images are not relative to one another. In contrast, the PGE_2_/AA ratio images are fitted to a common scale and can be compared to each other. White arrows point out exemplary lesion areas; red arrows indicate peri-lesional tissue with full myelination; scale bar: 5 mm. **b** PGE_2_ signal intensities were extracted as mean intensity per tissue and normalized to the internal standard of PGE_2_ to yield average detected concentration per pixel (µM/pixel). Each dot presents one donor. **c** Paired analysis of PGE_2_ levels and PGE_2_/AA ratio based on *broad* classification (one representative ROI) within MS tissues. **d-e** Paired analysis of PGE_2_ levels and PGE_2_/AA ratio based on PLP or HLA-DR classification (*in depth* classes). **f** Representative images of HLA-DR immunoreactivity in MS WM tissue; scale bar: 50 µm, zoom in: 10 µm. **g** Spearman correlation (r_s_) of PGE_2_/AA ratio values with HLA-DR MI in present, partial and absent PLP tissue categories. Data is shown as box plots with median ± quartiles; whiskers extend to minimum and maximum. Data have been statistically tested for three groups by Friedman test (paired) for non-normally distributed data and Dunn’s post-hoc analysis. Exact p-values are reported and statistical significance set at p<0.05 (red).

**Fig.4 Increased microglial EP2 expression in MS lesions**

**a** Schematic overview of PGE_2_ receptors EP1-4 and their G-protein coupled signaling pathways. EP2 (blue) and EP4 (yellow) are highlighted. **b** Representative cropped immunoblots of EP2, EP4 (upper panel each) and β-actin (lower panel) from whole brain tissue lysates of NNC and MS lesions. **c** Densitometric quantification of EP2 and EP4, normalized to β-actin in NNC (N=9) and MS lesions (N=14). **d** Representative images of EP2 (cyan), Iba1 (magenta), and TMEM119 (yellow) immunoreactivity in WM of NNC and mixed A/I MS lesions (peri-lesion and lesion rim). Panels show outlined excerpt at higher magnification; scale bar: 50 µm. **e** Quantification of EP2^+^ cells and EP2 mean fluorescent intensity within microglia (Iba1^+^TMEM119^+^ cells) in NNC and MS tissues (N=5). **f** *mRNA* expression of *PTGER2* (encoding EP2) and EP2 protein were measured in human iPSC-derived microglia (hiPSC microglia) non-stimulated (resting) or stimulated with LPS + IFNγ for 24 hrs (pro-inflam). Data is shown as box plots with median ± quartiles; whiskers extend to minimum and maximum. Data have been statistically tested for three groups by ordinary one-way ANOVA with Dunnetts correction or Kruskal-Wallis test for non-normally distributed data and Dunn’s post-hoc analysis. For two groups an unpaired student-t test with Welch’s correction was used when the variance of the groups were significantly different or Mann Whitney test for non-parametric datasets. Exact p-values are reported and statistical significance set at p<0.05 (red).

**Fig.5 PGE_2_ signaling adds to immunological response in microglia**

**a** Schematic overview of PGE_2_ and EP inhibitor treatment strategy on resting and pro-inflammatory hiPSC microglia. **b** Venn diagram of significantly differentially expressed genes (DEG) presenting up (top) and downregulated genes (bottom) in resting (green) vs. pro-inflammatory (blue) microglia treated with PGE_2_ vs. vehicle; N_TR_=5. **c** Volcano plot representing significant DEGs comparing PGE_2_ treatment vs. vehicle in resting microglia with respect to -log_10_ P adjusted in the y-axis and log_2_ fold change in the x-axis. **d** Volcano plot representing significant DEGs comparing PGE_2_ treatment vs. vehicle in pro-inflammatory microglia. **e** Volcano plot visualizing DEGs regulated by EP2i + PGE_2_ vs EP4i+ PGE_2_ in pro-inflammatory microglia. **f** mRNA expression of *CXCR4*, *PTGES*, *TREM2, CCL13* and *PAK1* in pro-inflammatory microglia treated with PGE_2_+EP inhibitors; N=3. **f** Over-Representation analysis of enriched KEGG pathways in proinflammatory iPSC microglia treated with PGE_2_ + EP2i vs. PGE_2_+EP4i. Data is shown as box plots with median ± quartiles; whiskers extend to minimum and maximum. Data have been statistically tested for four groups by Friedman test for non-normally distributed data and Dunn’s post-hoc analysis. Exact p-values are reported and statistical significance set at p<0.05 (red).
