# Supplementary material for: Spatial mapping of the AA-PGE2-EP axis in multiple sclerosis lesions
Source: Acta Neuropathol. 2025 Apr 29;149(1):39. doi: 10.1007/s00401-025-02878-3 (PMC12041062; doi:10.1007/s00401-025-02878-3)
Supplement: Supplementary file 8 — Supplementary file8 (DOCX 25 kb) [file 401_2025_2878_MOESM8_ESM.docx]

**Supplementary figure legends**

**Spatial mapping of the AA-PGE_2_-EP axis in multiple sclerosis lesions**

Cathrin E. Hansen^1,2,3*^, Julia Konings^1,2,3*^, Gabor Toth^5,6^, Serhii Chornyi^1^, Manon Karsten^1^, Bert van het Hof^1^, Susanne M.A. van der Pol^1^, Stephanie D. Beekhuis-Hoekstra^1^, Nine Kok^1^, Wing Ka Fung^1^, Naomi S. Dijksman^7^, Wia Baron^7^, Maarten E. Witte^1,2,3,4^, Ingela Lanekoff^5,6^, Helga E. de Vries^1,2,3^, Gijs Kooij^1,2,3,4#^.

^1^ Amsterdam UMC location Vrije Universiteit Amsterdam, Department of Molecular Cell Biology and Immunology, De Boelelaan 1117, Amsterdam, The Netherlands

^2^ Amsterdam Neuroscience, Amsterdam UMC, Amsterdam, The Netherlands

^3^ MS Center Amsterdam, Amsterdam UMC Location VU Medical Center, Amsterdam, The Netherlands

^4^Amsterdam Institute for Immunology and Infectious Diseases, Amsterdam UMC, Amsterdam, The Netherlands

^5^ Department of Chemistry, BMC, Uppsala University, 75237 Uppsala, Sweden

^6^Center of Excellence for the Chemical Mechanisms of Life, Uppsala University, Sweden

^7^Biomedical Sciences of Cells & Systems, Section Molecular Neurobiology, University of Groningen, UMCG, A. Deusinglaan 1, Groningen, The Netherlands

^*^ Both authors contributed equally to this work

^#^ Corresponding author: Gijs Kooij (email: g.kooij@amsterdamumc.nl, tel: +31 (0) 204448080)

**Supplementary Fig. 1 Vascularized area in relation to AA lipid levels in MS brain tissue and COX2 immunoreactivity**

**a** Representative image of UEA-I (vascular marker) in MS tissue; scale bar: 50 µm. **b** Quantification of vascular area [µm^2^] in full, partially demyelinated and demyelinated areas. **c** Spearman correlation (r_s_) of AA with the vascular area in PLP categories. **d** Representative image of COX2, CD45 (panel 2) and Collagen IV (Coll IV, vascular marker) immunoreactivity in MS WM tissue. Panel 1 indicates CD45^+^ cells with COX2 immunoreactivity distant to the vasculature. Panel 2 emphasizes COX2 expression in CD45^+^ perivascular immune cells; scale bar: 50 µm, zoom in: 10 µm. **e** Confocal images of COX2 and CD45 visualized as orthogonal view to emphasize (peri)nuclear and cytoplasmic COX2 expression; scale bar: 5 µm.

**Supplementary Fig. 2 PGE2/AA and HLA-DR distribution in MS tissue**

Representative ion images of PGE_2_/AA ratio and corresponding HLA-DR staining in MS brain tissue. White arrows indicate MS lesions.

**Supplementary Fig. 3 Ptger4/EP4 expression in iPSC microglia**

**a** *mRNA* expression of *ptger4* (encoding EP4) and **b** EP4 protein were measured in human iPSC-derived microglia (hiPSC microglia) control (resting) or stimulated with LPS + IFNγ for 24 hrs (pro-inflam). Data is shown as box plots with median ± quartiles; whiskers extend to minimum and maximum.

**Supplementary Fig. 4 PCA of resting and pro-inflammatory hiPSC microglia treated with vehicle, PGE_2_ and PGE_2_+/- EP2/4 inhibitor**

The first two principal components of bulk RNA sequencing of treated resting (green) and pro-inflammatory (blue) hiPSC microglia. **a** The principal component analysis (PCA) showed a clear separation between the vehicle, PGE_2_ and PGE_2_+EP2i treated microglia under resting and pro-inflammatory (**b**) conditions, while PGE_2_+EP4i only differs under pro-inflammatory conditions, N_TR_=5.

**Supplementary Fig. 5 Quantification of TMEM119 intensity in Iba1^+^ cells from MS lesions comparing EP2^+^and EP2^-^ populations**

Quantification of TMEM119 intensity in EP2^-^ and EP2^+^ cells within Iba1^+^ cells in MS lesions (N=5). Data have been statistically tested with a paired student-t test. Exact p-values are reported and statistical significance set at p<0.05 (red).
